# Supplementary material for: Overestimation in angular path integration precedes Alzheimer’s dementia
Source: Curr Biol. 2023 Nov 6;33(21):4650–4661.e7. doi: 10.1016/j.cub.2023.09.047 (PMC10957396; doi:10.1016/j.cub.2023.09.047)
Supplement: Document S1. Figures S1–S6, Table S1, and supplemental references [file mmc1.pdf]

Current Biology, Volume *33*

Supplemental Information

Overestimation in angular path integration precedes Alzheimer's dementia

Andrea Castegnaro, Zilong Ji, Katarzyna Rudzka, Dennis Chan, and Neil Burgess

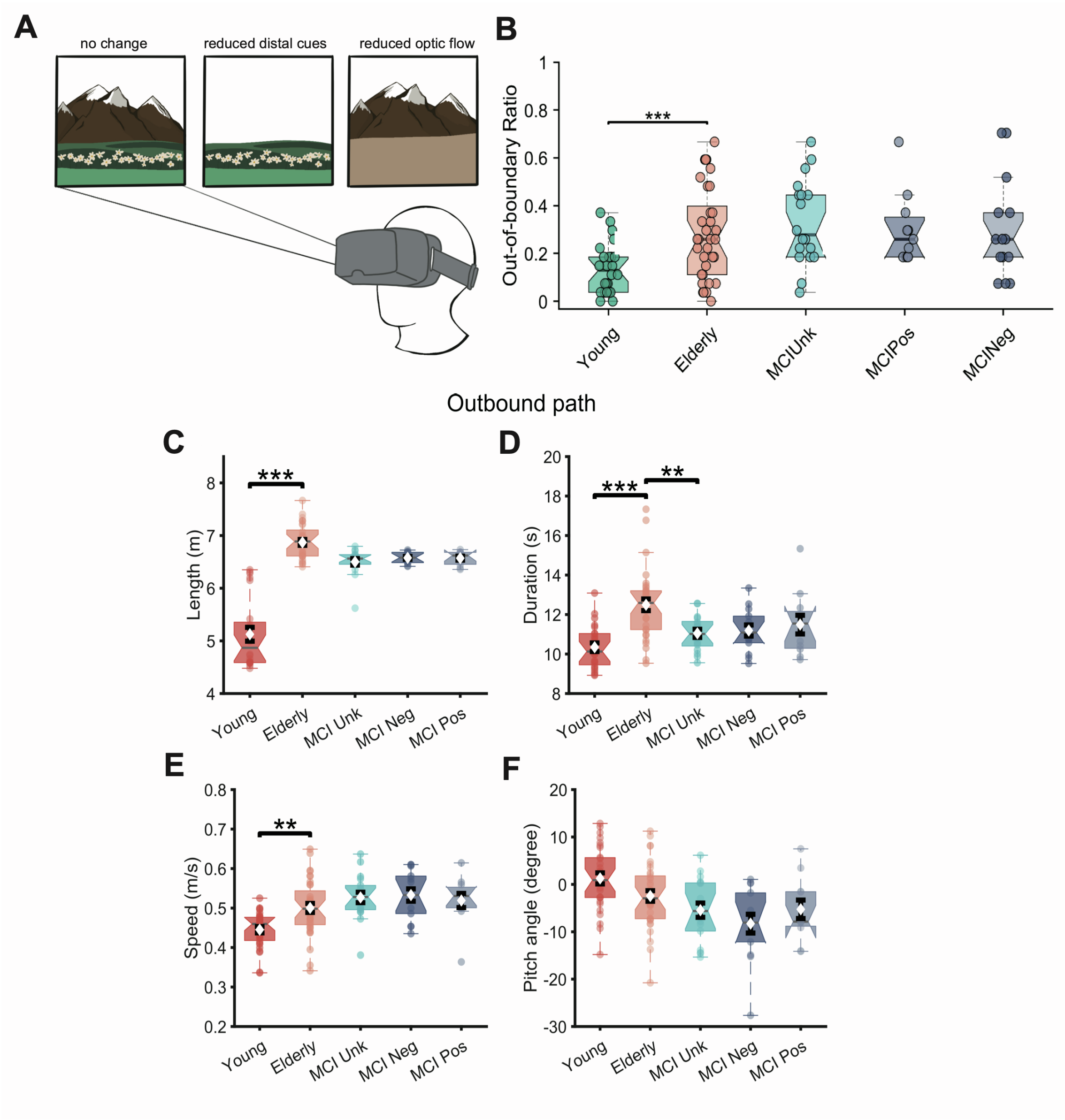

Figure S1: Experimental setup and behavioral results. Related to Figure 1 and STAR Methods. *(A) Inbound path environmental conditions. Following completion of the outbound path, participants were presented with three distinct environmental conditions in a randomized order just prior to starting the return inbound path. Environmental conditions were applied with a fading to black transition in the virtual reality view. The "No Change" condition consisted of an unchanged environment as compared to the outbound path. The "Reduced Distal Cues" condition involved the removal of distant landmarks from the scene. The "Reduced Optic Flow" condition involved the removal of details from the ground plane and replacement with a solid-colored plane merging seamlessly with the distant cues. (B) out-of-boundary ratios across groups. A two-way ANOVA analyzed the effect of environmental conditions (no change, reduced optic flow, reduced distal cues) and participant group (young vs. healthy elder participants vs. MCIUnk vs. MCIPos vs. MCINeg patients) on the out-of-boundary ratios. There was no significant interaction effect (F(8,285)=0.29, p=0.931), no significant environmental effect (F(2,285)=0.21, p=0.936). There was a significant group effect with F(4,285)=8.64, and p<0.001. Post-hoc Bonferroni corrected comparisons showed that the young group made significantly less out-of-boundary trials than the other four groups: young vs elderly p<0.001; young vs MCIUnk p<0.001; young vs MCIPos p<0.001; young vs MCINeg p=0.002. (C-F) outbound path information for different groups. (C) average length of the outbound path (sum of* l_1 *and* l_2*). (D) average time that to complete the outbound path. (E) reconstructed average walking speed during the outbound path. Each dot represents the average value of the participant. (F) average pitch angle obtained from the headset orientation. Filled diamonds represent the group mean with black error bars indicating the standard error of the mean (SEM). The indent and gray horizontal bar in each box represents the median value. The bottom and top of each box*

*represent the 25th and 75th percentiles, respectively. Horizontal bars indicate significant post-hoc comparisons. Differences reported are between young and healthy older and between healthy older and any of the MCI groups. The significance of comparisons between young and MCI subgroups were not marked on figures for legibility and are not referred to in the main text. * ($p<0.05$), **($p < 0.01$), ***($p < 0.001$) are marked from the two-way ANOVA with post hoc multiple comparisons with Bonferroni correction.*

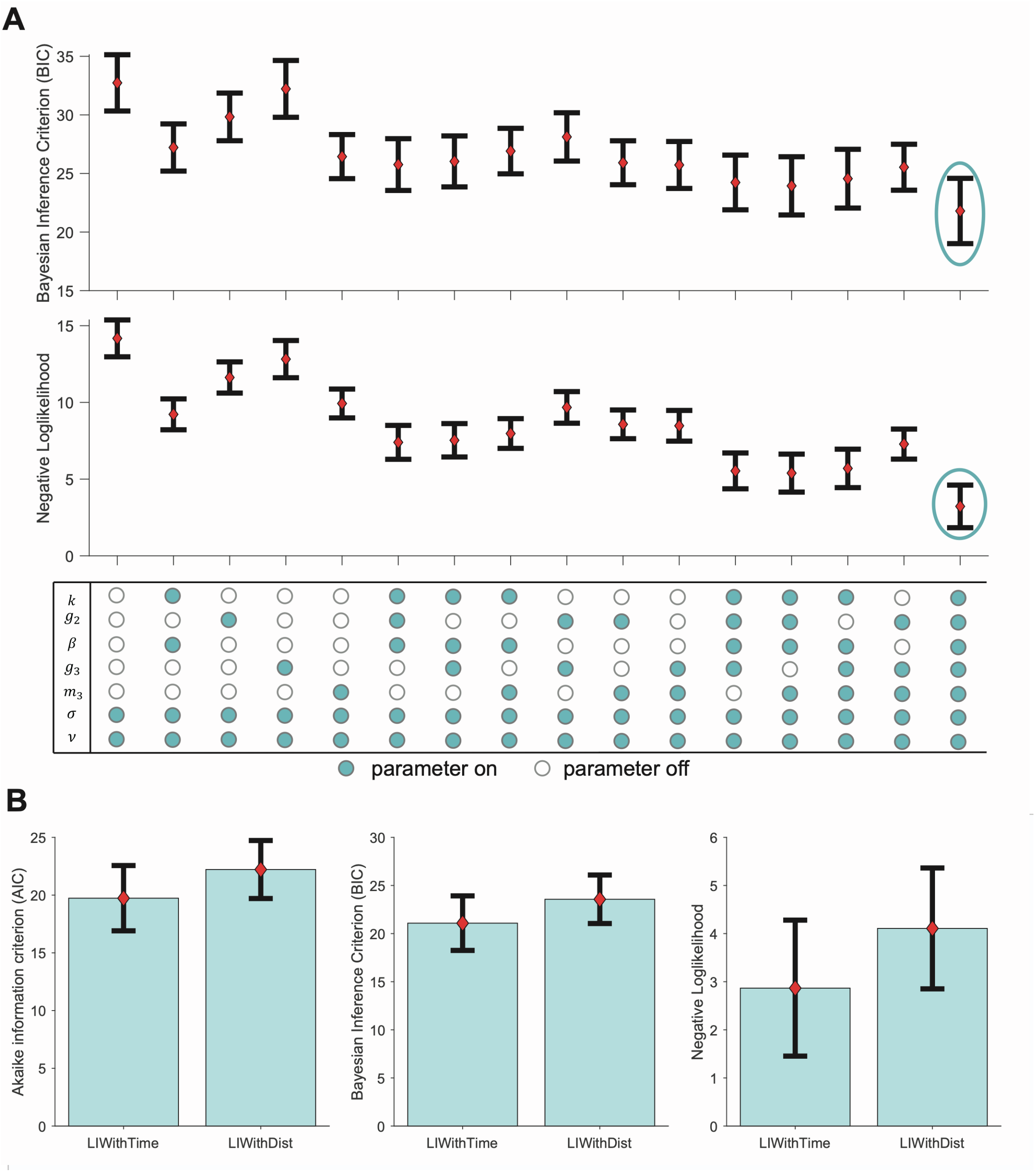

Figure S2: GLAMPI model selection. Related to Figure 2. *(A) Comparison between different configurations of the model and the relative Bayesian Information Criterion (BIC) and negative loglikelihood value (NLL). Top: BIC values (mean with SEM) of candidate models. Middle: NLL values (mean with SEM) of candidate models. Bottom: blue/white circles represent considered/unconsidered parameters. Candidate models were created from all different combinations of models varying type and the number of source errors used. The selected model with lowest BIC or NLL values are circled in blue. (B) comparison between leaky integration over time and leaky integration over distance. It shows the model performance of AIC BIC and loglikelihood of the two models on the data from healthy elderly participants. Leaky integration of distance is described in the section '*Relationship with the leaky-integration model used in desktop VR*'. We found that leaky integration over time has a better performance than leaky integration over distance, reflected in the lower AIC BIC and negative Loglikelihood values. However, the Wilcoxon rank sum test shows that the AIC, BIC and negative Loglikelihood values are not significantly different from each other in these two models.*

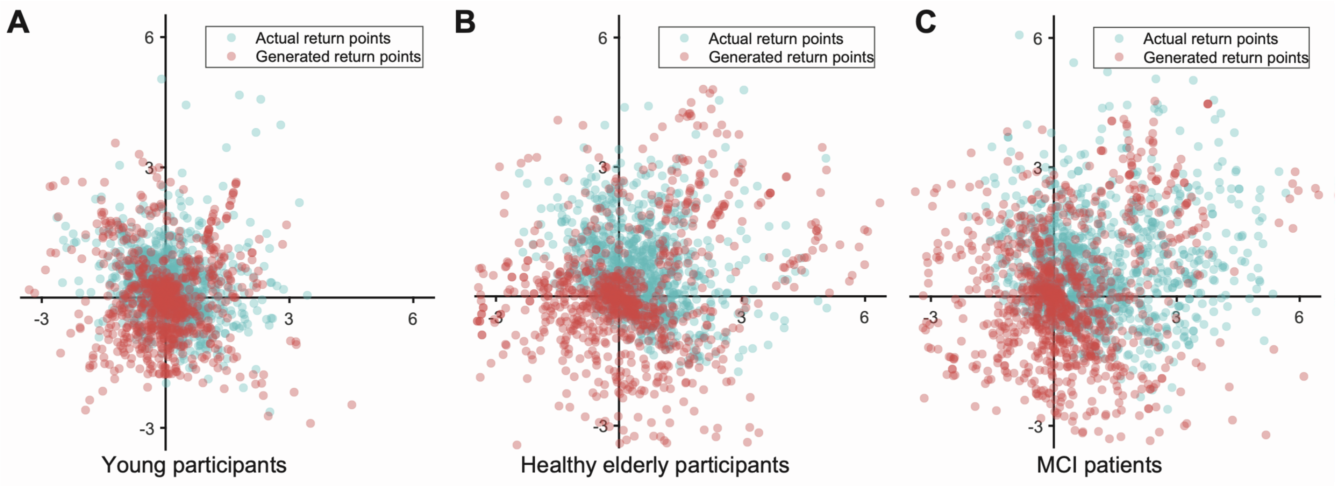

Figure S3: Summarized actual return locations and generated return locations. Related to Figure 3. *Showing young participants, healthy elderly participants, and MCI patients. Blue dots: actual return locations (each for one trial). Red dots: predicted return locations from the model.*

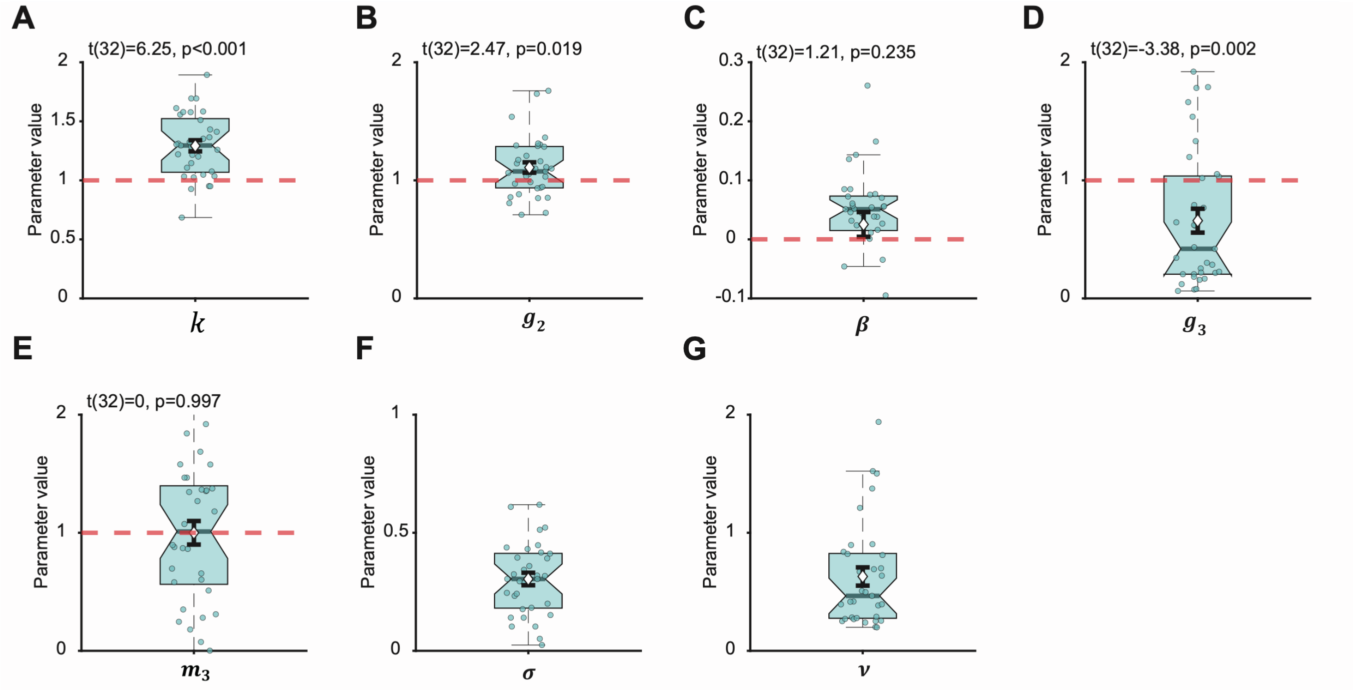

Figure S4: Estimated parameters of the GLAMPI model on the data from healthy elderly participants. Related to Figure 4. *Showing the significance of each parameter's difference from its optimal value (red dashed line) at which no error would occur according to one sample two-tailed t-test results. Each dot represents a parameter estimated from the data of one participant (averaged across three conditions). Box plots shown as in Figure S1.*

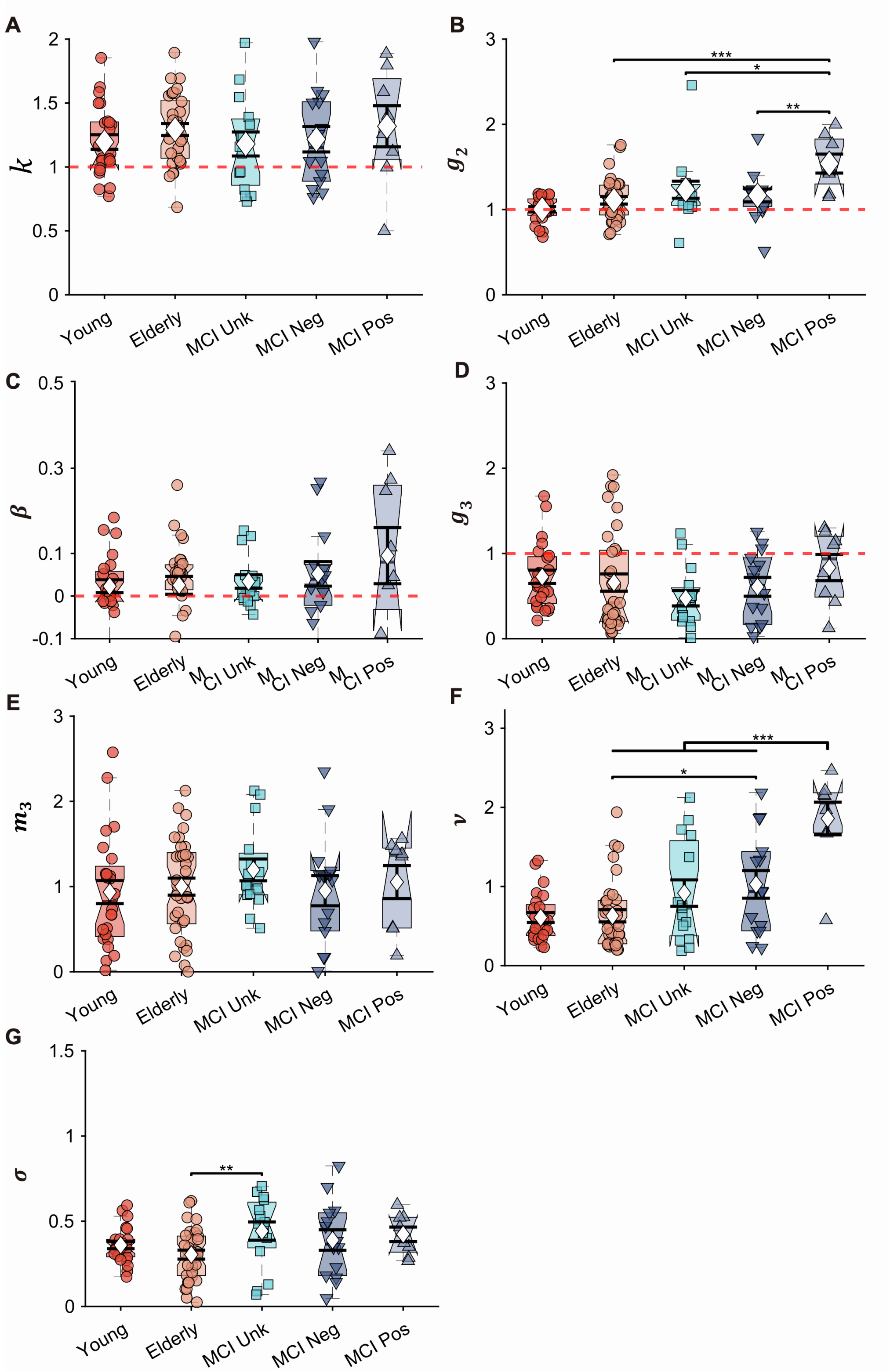

Figure S5: GLAMPI parameter comparisons among young, healthy elderly participants, MCI unknown, MCI negative and MCI positive. Related to Figure 4. *Two-way ANOVA on the GLAMPI parameters with environmental condition and group as factors. Each dot represents a parameter estimated from the data of one participant (averaged across three conditions). In each plotted box, the white diamond marks the mean, the black bars mark the standard error of the mean (SEM) and the grey horizontal bar marks the median. The bottom and top of each box mark the 25th and 75th percentiles, respectively. Observations beyond the whisker length are outliers. Red dashed lines (reference lines) mark the optimal parameter value (i.e., no error occurs at this value). The significance of comparisons between young and MCI subgroups were not marked on figures for legibility and because these contrasts are not referred to throughout the manuscript. * (p<0.05), **(p < 0.01), ***(p < 0.001) are marked from the two-way ANOVA with post hoc multiple comparisons with Bonferroni correction.*

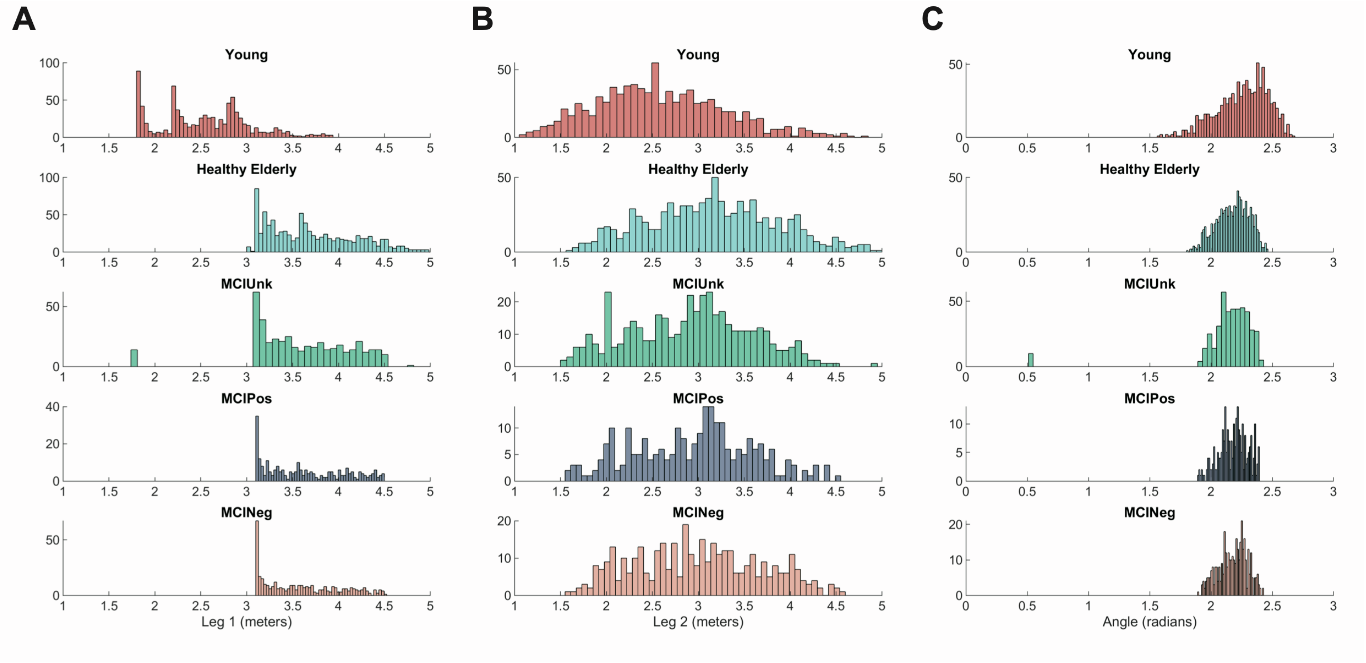

Figure S6: Histograms of the outbound paths for each group. Related to STAR Methods. *(A) The distribution of* l_1*; (B) The distribution of* l_2*; (C) The distribution of the turning angle (*θ_2*) between* l_1 *and* l_2*.*

Group	Sample Size	Age (Mean ± SD)	Sex (Female %)	Years in Ed (Mean ± SD)	ACE-R (Mean ± SD)	MMSE (Mean ± SD)
Young	31	21.16 (3.17)	23 (74.19%)	14.77 (1.21)		
Elderly (controls)	36	68.31 (7.19)	26 (69.44%)	15.08 (3.74)	97.21 (3.29)	29.74 (0.57)
MCI Negative	11	71.29 (9.09)	4 (28.57%)	14.54 (4.41)	86.69 (7.62)	27.62 (2.66)
MCI Positive	14	75.73 (7.30)	3 (27.27%)	14.45 (4.01)	82.82 (10.04)	25.82 (5.21)
MCI Unknown	18	71.22 (8.28)	7 (38.89%)	14.33 (3.43)	89.71 (5.43)	28.41 (1.58)

Table S1: Demographics and neuropsychological tests (where collected). Related to STAR Methods. *Reported data are means and standard deviations. Neuropsychological tests reported are the Addenbrooke's Cognitive Examination-Revised (ACE-R)*[S1] *and the Mini Mental State Examination (MMSE)*[S2]*. We carried out two-sample tests to check if there is any difference in the two demographic variable between MCI+ and MCI- patients. It showed that there is no significant age difference between these two groups (t(23)=0.200), and no significant difference in educational duration (t(22)=0.962). Note that all data was been tested for normality with the Anderson-Darling test before carrying out the t-test.*

Supplemental References

S1. Mioshi, E., Dawson, K., Mitchell, J., Arnold, R. & Hodges, J. R. The Addenbrooke's Cognitive Examination revised (ACE-R): A brief cognitive test battery for dementia screening. *Int J Geriatr Psychiatry* **21**, 1078–1085 (2006).

S2. Folstein, M. F., Folstein, S. E. & McHugh, P. R. 'Mini-mental state'. A practical method for grading the cognitive state of patients for the clinician. *J Psychiatr Res* **12**, 189–198 (1975).
