# Supplementary material for: Overestimation in angular path integration precedes Alzheimer’s dementia
Source: Curr Biol. 2023 Nov 6;33(21):4650–4661.e7. doi: 10.1016/j.cub.2023.09.047 (PMC10957396; doi:10.1016/j.cub.2023.09.047)
Supplement: Document S2. Article plus supplemental information [file mmc2.pdf]

Current Biology

Article

Overestimation in angular path integration precedes Alzheimer's dementia

Highlights

- Specific aspects of path integration are impaired early in Alzheimer's disease (AD)
- A generative model specifically implicates angular encoding gain and angular noise
- Angular path integration impairment in early AD is not an extension of healthy aging

Authors

Andrea Castegnaro, Zilong Ji, Katarzyna Rudzka, Dennis Chan, Neil Burgess

Correspondence

n.burgess@ucl.ac.uk

In brief

Path integration is impaired early in Alzheimer's disease (AD). Castegnaro, Ji, et al. present a computational model of the processes underlying generation of the return path, which indicates that, among patients with mild cognitive impairment, those with biomarkers for AD have a specific increase in angular encoding gain and angular noise.

Castegnaro et al., 2023, Current Biology *33*, 4650–4661
November 6, 2023 © 2023 The Author(s). Published by Elsevier Inc.
https://doi.org/10.1016/j.cub.2023.09.047

CellPress

CellPress
OPEN ACCESS

Current Biology

Article

Overestimation in angular path integration precedes Alzheimer's dementia

Andrea Castegnaro,[1,2,4] Zilong Ji,[1,3,4] Katarzyna Rudzka,[1] Dennis Chan,[1] and Neil Burgess[1,2,5,*]
[1]UCL Institute of Cognitive Neuroscience, University College London, 17 Queen Square, London WC1N 3AZ, UK
[2]UCL Queen Square Institute of Neurology, University College London, Queen Square, London WC1N 3BG, UK
[3]Peking-Tsinghua Center for Life Sciences, Academy for Advanced Interdisciplinary Studies, Peking University, Haidian District, Beijing 100871, China
[4]These authors contributed equally
[5]Lead contact
*Correspondence: n.burgess@ucl.ac.uk
https://doi.org/10.1016/j.cub.2023.09.047

SUMMARY

Path integration (PI) is impaired early in Alzheimer's disease (AD) but reflects multiple sub-processes that may be differentially sensitive to AD. To characterize these sub-processes, we developed a novel generative linear-angular model of PI (GLAMPI) to fit the inbound paths of healthy elderly participants performing triangle completion, a popular PI task, in immersive virtual reality with real movement. The model fits seven parameters reflecting the encoding, calculation, and production errors associated with inaccuracies in PI. We compared these parameters across younger and older participants and patients with mild cognitive impairment (MCI), including those with (MCI+) and without (MCI−) cerebrospinal fluid biomarkers of AD neuropathology. MCI patients showed overestimation of the angular turn in the outbound path and more variable inbound distances and directions compared with healthy elderly. MCI+ were best distinguished from MCI− patients by overestimation of outbound turns and more variable inbound directions. Our results suggest that overestimation of turning underlies the PI errors seen in patients with early AD, indicating specific neural pathways and diagnostic behaviors for further research.

INTRODUCTION

"Path integration" (PI), updating your estimated location and orientation using self-motion signals, including optic flow and vestibular and proprioceptive feedback, is a crucial ability of mobile animals. Impaired PI is a sensitive and specific behavioral marker of pre-dementia of Alzheimer's disease (AD), manifesting in mild cognitive impairment (MCI)[1] and specifically in those cases attributable to AD.[2] Furthermore, entorhinal cortex, a brain region whose spatially modulated "grid cells" are thought to underpin PI[3,4] in rodents[5] and humans,[6] is one of the first regions to manifest AD neuropathology.[7] Indeed, young people with increased genetic risk of AD have disrupted grid-like representations in entorhinal cortex[8,9] (reviewed by Segen et al.[10]).

PI could, therefore, provide a sensitive and specific test of early AD. Here, we attempted to identify the underlying components of PI in healthy older adults and find out which components are disrupted in MCI patients with biomarkers for AD in their cerebrospinal fluid (CSF).[2] Being able to assess different components of PI might allow creation of more precise cognitive tests for the presence of dementia at earlier stages and metrics with higher diagnostic sensitivity than previously used.

"Triangle completion" is a simple test of PI that shows impairments prior to the onset of typical symptoms in Alzheimer's dementia.[1,2] In this task, participants are guided on two straight legs joined by a turn (outbound path) and are then asked to return to the start location guided by memory rather than any environmental cues (inbound path; Figures 1A and 1B). Behavioral studies have categorized errors in "encoding,"[11–13] "calculation,"[14,15] and "production."[16,17] Encoding errors accrue in the internal representation of self-motion information during the outbound path. Calculation errors refer to integrating the encoded information to determine the intended inbound path toward the start location. Finally, production errors occur when translating the intended inbound path into physical action.

Harootonian et al.[13] found errors corresponding to the first leg being given a smaller magnitude than the second leg when calculating the return path by vector addition of the outbound legs. This is consistent with the idea of leaky integration of velocity over time, which continues until the return path is calculated, resulting in greater overall reduction of the first leg than the second leg, which we consider as a calculation error rather than purely an encoding error. Leaky integration of velocity can also explain the tendency to undershoot the goal location when reproducing distances or completing a triangle. In addition, when participants experience a sequence of trials in triangle completion experiments, responses in preceding trials produce a regression-to-the-mean-like effect on responses in the current trial.[18] These effects are considered production errors (see also Harris et al.[19] and Harootonian et al.[13]) and can also be seen in angle and distance reproduction tasks that dissociate the size of the encoded and produced rotations and distances.[17]

 © 2023 The Author(s). Published by Elsevier Inc.
This is an open access article under the CC BY license (http://creativecommons.org/licenses/by/4.0/).

CellPress
OPEN ACCESS

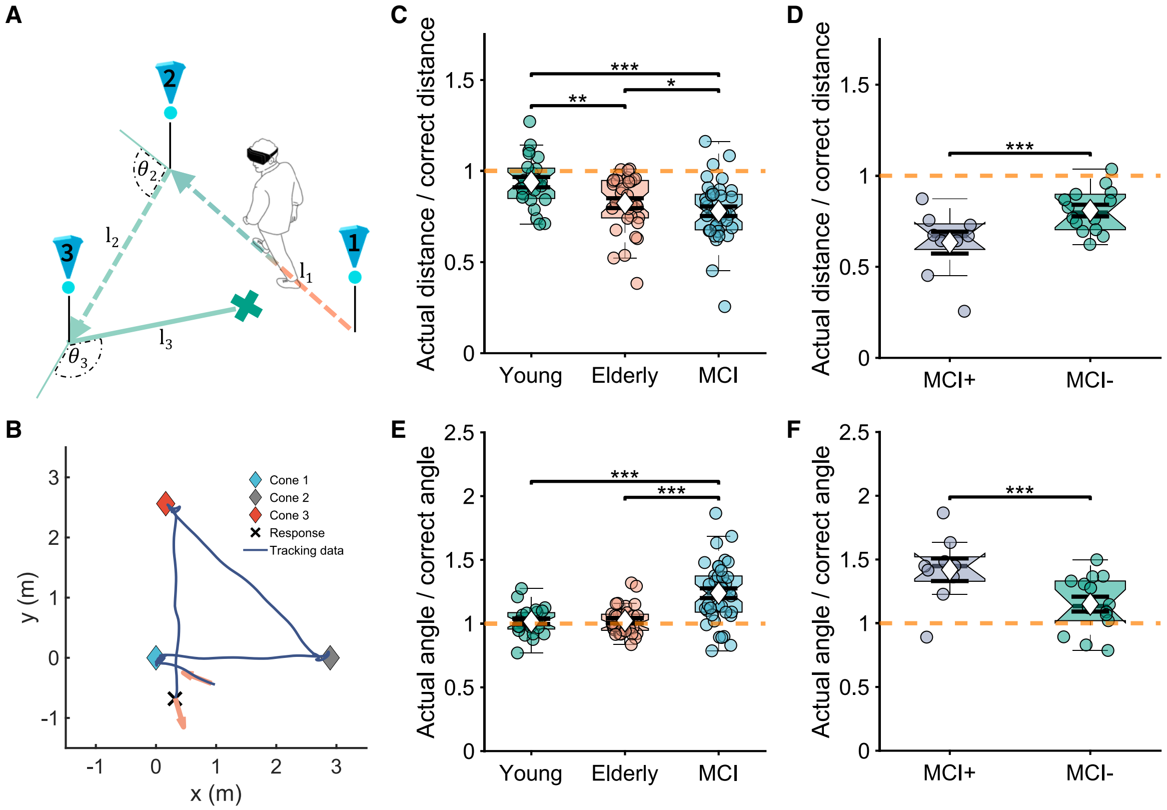

Figure 1. Performance of the triangle completion task

(A) Participants completed the task in immersive virtual reality (iVR), making real-world movements while wearing iVR googles. Participants were guided by visible cones displayed one at a time through two outbound legs (l1 and l2) joined by a turn (θ_2). Each cone disappeared immediately upon the participant colliding with it, and only then, a cone appeared at the next location. At the response stage, no cones or other guidance were available except temporary in-VR text instructing the participants to return to the remembered location of cone 1 (inbound path; via an angle θ_3 and a distance l_3). The cross indicates the participant's response (their final location). See also Figure S1.

(B) Walked path from a single trial of a young participant reconstructed from the pre-processed tracking data and aligned to the x axis. Arrows indicate the starting and final head direction.

(C and D) Proportional linear distance error for the return path across groups.

(E and F) Proportional angular error for the return path across groups. Each dot represents the estimated data of one participant (averaged across three conditions). In each box, the white diamond marks the mean, the black bars mark the standard error of the mean (SEM) and the indent and gray, horizontal bar marks the median. The bottom and top of each box mark the 25th and 75th percentiles, respectively. Observations beyond the whisker length are outliers. Red dashed lines mark the optimal parameter value (i.e., no error occurs at this value). *$p < 0.05$, **$p < 0.01$, and ***$p < 0.001$ are marked from a two-way ANOVA with post hoc multiple comparisons with Bonferroni correction. Please refer to Figure S1 for additional information about the task conditions and additional information about the outbound path for different groups.

Successful navigation relies on accurate updating of spatial representations via the integration of both linear and angular self-motion information derived from optic flow, proprioception, motor efference copy, and vestibular feedback. There are separate components in the vestibular system to detect these different movement elements: the otoliths for linear motion and semi-circular canals for the angular motion.[20] Although the coding of linear speed has been reported in the speed cells of the entorhinal cortex[21] and medial septum,[22] separate populations of cells appear to encode angular velocity.[23–25] Grid cells are thought to mediate PI[4,26–28]; however, it is not yet clear how they integrate angular velocity, because the head-direction coding observed in medial entorhinal cortex cells (including conjunctive grid cells[29]) is not a viable proxy for the direction of movement required for PI.[30] The possibility of separate inputs to PI is reflected in our choice to dissociate the linear and angular components of behavior (see discussion for more details).

Here, we present a novel generative model of the inbound path in triangle completion performed in immersive virtual reality (iVR) with real movement. We parametrized potential effects of encoding, calculation, and production errors in linear and angular components separately in our generative linear-angular model of PI (GLAMPI). We validated the model by fitting the behavior of healthy elderly participants, testing candidate models comprising all combinations of the sources of error suggested by the studies referred to above. The best fitting model included the main features of all previous models, approximated using a minimal number of parameters for simplicity and considering data constraints. We then searched for systematic differences in the parameters fitting data from different populations, including healthy

younger and older participants and patients with MCI. Finally, we looked for systematic differences in parameters within the MCI group between subgroups with (MCI+) or without (MCI−) CSF biomarkers, indicating the presence of underlying Alzheimer's neuropathology.

A basic analysis of the performance of the patients and healthy older participants (their age and IQ matched controls) was reported in Howett et al.[2] Here, we use the generative model to interpret behavioral performance in terms of more specific underlying mechanisms. Additionally, we include data from a young control population and contrast them with the healthy elderly to distinguish any impairment specific to AD pathophysiology from an extension of healthy aging deficits. We also adjusted our analysis of trials in which return paths were very short or went out of bounds to reflect our interest in PI mechanisms (see the data pre-processing section).

Our goal is to provide a better understanding of the component processes underlying PI and to contribute to the development of future navigation-based tools aimed at AD diagnosis.

RESULTS

Participant groups comprised healthy young, healthy elderly, and patients with MCI. MCI patients were further categorized into subgroups MCI+ (positive biomarker), MCI− (negative biomarker), and MCI unknown (biomarker status unknown) based on CSF tests for the beta-amyloid and tau biomarkers of AD neuropathology. MCI+ patients are considered to have pre-dementia AD, whereas MCI− patients include a heterogeneous variety of non-AD causes of cognitive impairment, including mood and sleep disorders. Each participant completed the triangle completion task in iVR, making real-world movements while wearing iVR googles. In each trial, participants walked a two-legged outbound path, guided by numbered cones (see Figure 1 and STAR Methods). The inbound return path to the starting position was performed under three environmental conditions (Figure S1): (1) no change (unchanged virtual environment), (2) reduced optic flow (ground details were replaced by a plain texture), and (3) reduced distal cues (the surrounded mountains were temporarily removed). All landmarks were too distant to provide any indication of location. Participants completed nine trials (or 12 for the young group) within each of the three environmental conditions, totaling 27 (or 36 for the young group) trials per participant.

PI behavioral performance across groups

Differences in performance between groups were assessed using two-way ANOVAs (on the proportional distance and angular errors separately) with factors group and environmental condition (see STAR Methods for details) and post hoc multiple comparisons with Bonferroni correction.

Two-way ANOVA on young, healthy elderly, and pooled MCI groups revealing no interaction between group and environmental condition ($F(4,278) = 0.0$, $p = 1.00$) and no main effect of environmental condition ($F(2,278) = 0.0$, $p = 0.999$) on proportional distance error. A main effect of group was found ($F(2,278) = 15.96$, $p < 0.001$). Post hoc comparisons showed that the MCI group under-walked the distance to the start location (proportional distance error: 0.77 ±0.19; Figure 1C) more than healthy elderly (0.82 ±0.15; $p = 0.022$) and young groups (0.94 ± 0.14; $p < 0.001$), the healthy elderly under-walked more than the young group ($p = 0.007$), and all three groups under-walked (two-tailed t test on proportional linear distance error $\neq 1$; $t(23) = -2.15$ for young group, $t(32) = -6.53$ for healthy elderly, and $t(38) = -8.43$ for pooled MCI; all $p < 0.05$). Given that young participants completed more trials (see the path integration task section for details), a secondary analysis considered only the first nine trials for this group for each condition. Although the main group effect and the group differences between young and elderly remained unchanged, the under-walking by young participants for these nine trials was no longer significant in the two-tailed t test ($t(23) = -1.76$, $p = 0.092$).

ANOVA of the proportional angular error showed a similar pattern, with no interaction ($F(4,279) = 0.0$, $p = 1.00$), no main effect of environmental condition ($F(2,279) = 0.0$, $p = 1.00$), and a main effect of group ($F(2,279) = 46.91$, $p < 0.001$). Post hoc comparisons revealed that the MCI group overturned toward the start location (proportional angular error: 1.26 ± 0.26; Figure 1E) more than healthy elderly (1.02 ± 0.11; $p < 0.001$) and young groups ($m = 1.02$ ±0.11; $p < 0.001$). Despite all groups over-turning, this only reached significance in the MCI group (two-tailed t test on proportional angular error $\neq 1$; $t(23) = 0.74$, $p = 0.469$ for young group, $t(32) = 1.19$, $p = 0.243$ for healthy elderly, $t(38) = 6.25$, $p < 0.001$ for pooled MCI).

ANOVA of the MCI+ vs. MCI− groups on the proportional distance error showed only a main effect of group ($F(1,63) = 38.65$, $p < 0.001$), post hoc comparisons revealed that MCI+ under-walked the distance to the start location (0.63 ±0.25; Figure 1D) more than the MCI− group (0.81 ± 0.12; $p < 0.001$), with both groups under-walking (two-tailed t test on proportional distance error $\neq 1$; $t(8) = -6.11$, $p < 0.001$ for MCI+; $t(13) = -5.98$, $p < 0.001$ for MCI−). ANOVA on the proportional angular error showed only a main effect of group ($F(1,63) = 9.40$, $p = 0.003$), with MCI+ over-turning (1.46 ± 0.26; Figure 1F) more than the MCI− group (1.15 ± 0.22; $p = 0.003$ with Bonferroni correction) and both groups over-turning (two-tailed t test on proportional angular error $\neq 1$; $t(8) = 4.72$, $p = 0.002$ for MCI+; $t(13) = 2.58$, $p = 0.023$ for MCI−).

Given the incidence of out-of-bound trials in our dataset, we reported the ratio of out-of-bound trials for each participant and conducted a two-way ANOVA to check for differences between groups (see Figure S1B for details). There were no significant differences between any pair of groups of healthy elderly participants, MCI unknown, MCI+ and MCI− patients. There were fewer out-of-bound trials in younger vs. older healthy participants ($F(4,285) = 8.64$; $p < 0.001$).

Generative model of the inbound paths in healthy elderly participants

To better understand the sources of errors made in the triangle completion task, we built a generative model of the angle turned and distance walked on the inbound path. We explicitly modeled encoding, calculation, and production errors that might contribute to overall error (Figure 2A).

(1) Encoding of the walked distances (l_1 and l_2) and turned angle (θ_2) into participants' mental space, represented by l'_1 and l'_2, and θ'_2, respectively, reflects a speed gain term k, and an angular gain term g_2 (see STAR Methods for details).

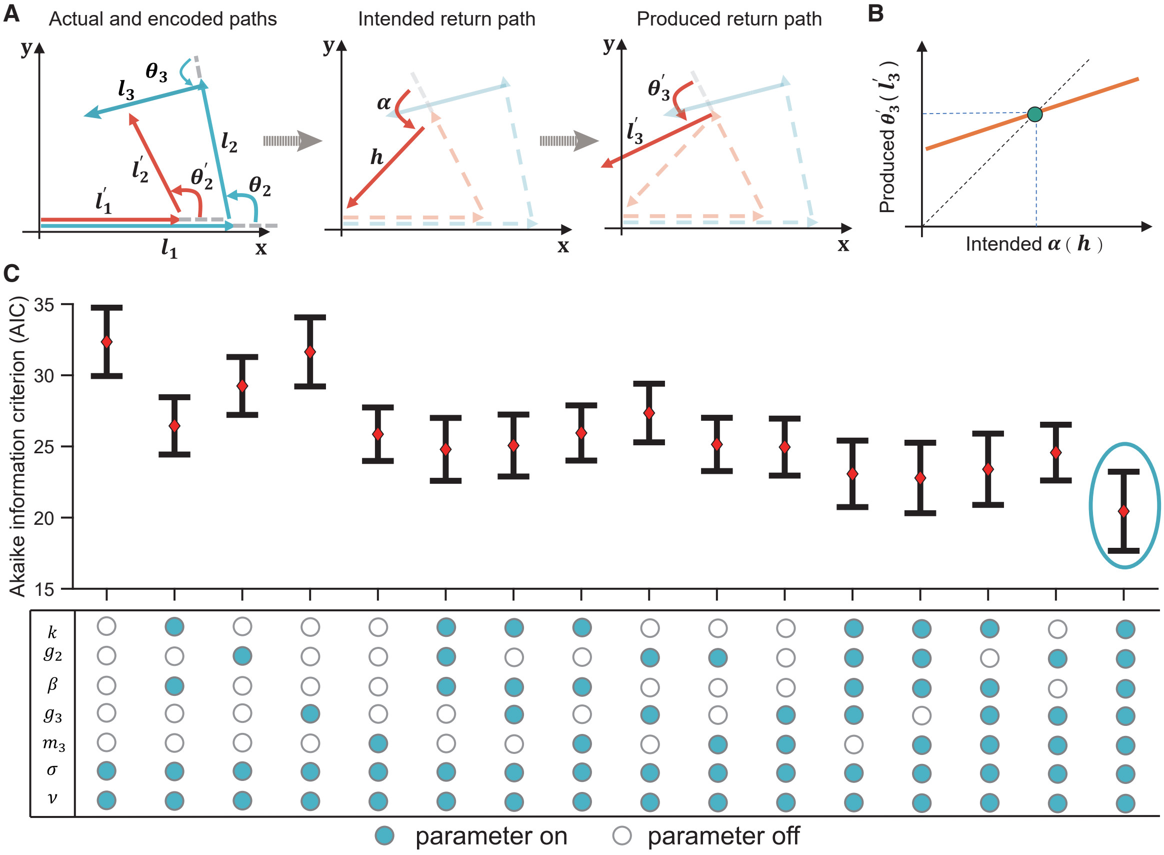

Figure 2. A generative linear-angular model of path integration (GLAMPI)

(A and B) (A) Blue arrows show the two legs of the actual outbound path and the return path. Red arrows show the representation of the outbound path (left) and the intended return path (middle), including encoding and calculation errors. The produced return path (right) includes production errors, including regression-to-the-mean angle/distance produced on other trials, as shown in (B) where the green point marks the participant's mean return angle/distance.

(C) Comparison among candidate models fitting data from the healthy elderly, comprising different combinations of potential sources of error (from the top): speed gain, angular gain, distance leak, distance regression slope, angular regression slope, distance noise, and angular noise (represented below: blue/white circles indicating considered/unconsidered sources of errors, respectively). The GLAMPI was selected as the one with the lowest Akaike information (mean with SEM shown), circled.

See also Figure S2.

(2) Calculation of the intended inbound distance h and angle α includes the integration over time of speed of walking along the two legs of the outbound path, which we assume can be leaky, with "leak" or "forgetting" term β. Other forms of calculation error are included in the Gaussian noise term added to the inbound distance and direction, described below.

(3) Production errors executing the intended walking distance h or turning angle α include the influence of return paths made in other trials of the testing session, producing a tendency for the return angle and distance to be biased toward the mean return angle and distance for the session (regression to the mean).[18] We model these errors in inbound distance and angle (l'_3 and θ'_3, respectively) using parameters m_3 and g_3, for the distance and angle regression slopes and the means of produced inbound distances and angles for that participant and condition, $\bar{l}_r$ and $\bar{\theta}_r$, respectively. Specifically, for distance production: $l'_3 = m_3 h + (1 - m_3)\bar{l}_r$, so that production is veridical if $m_3 = 1$ and biased toward the mean return distance $\bar{l}_r$ for $m_3 < 1$, and, similarly, for angular production (see Equation 5 in STAR Methods).

Finally, we capture other (unknown) sources of error by including noise in the actual inbound distance l_3 and angle θ_3, generated by sampling from two Gaussian distributions centered on l'_3, θ'_3 with variance σ, ν.

To find the model that best captures the behavior of healthy elderly participants with the minimal set of parameters, we compared candidate models comprising combinations of the above sources of error, starting with the fewest parameters and systematically incrementing complexity by adding new parameters (see Figure 2C), evaluating model complexity and fit to the data with the Akaike information criterion (AIC) (see Figure S2A for the Bayesian information criterion (BIC) and negative log-likelihood values). This process indicated that all identified parameters were necessary (Figure 2C), supporting the presence of all of these suggested types of error. We call this new model the GLAMPI hereafter.

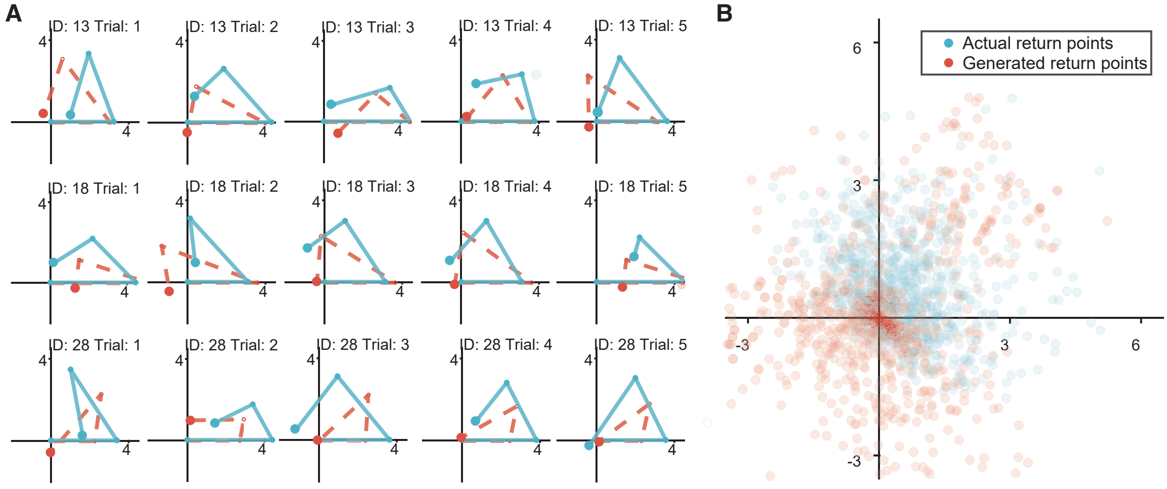

Figure 3. Actual and model-generated paths from healthy elderly participants

(A) Actual paths (blue, projected to straight lines) and model-generated paths (red dashed lines) from the first five trials under the no-change condition in three randomly selected participants. In both cases (blue and dashed red), the first outbound paths are projected onto the x axis.

(B) Summarized actual return locations (blue dots) and generated return locations (red dots) from all trials in all healthy elderly participants.

See Figure S3 for all groups.

The actual return locations and those generated by the model show a large overlap (see Figure 3 and Figure S3 for results on young participants and MCI patients). The GLAMPI did not find significant underestimation of the walked outbound distance (which would be represented by parameter $\beta > 0$); two-tailed t test with t(32) = 1.21, p = 0.235), suggesting that errors in healthy elderly are not explained by leaky integration of distance (Figure 4C). However, they did show significant gain in encoding the angle between the two legs of the outbound path (two-tailed t test $g_2 \neq 1$; t(32) = 2.47, p = 0.019; see Figure S4B) and regression to the mean in producing the inbound turn angle ($g_3 < 1$; two-tailed t test with t(32) = −3.38, p = 0.002; see Figures 4D and S4D) but not the inbound distance ($m_3 \neq 1$; two-tailed t test on t(32) = 0.00, p = 0.997; see Figures 4E and S4E).

Sources of PI errors in MCI

We next sought to identify which types of error were increased in MCI patients (Figures 4A–4G). First, two-way ANOVAs were performed on the GLAMPI parameters[31] with the factors environmental conditions (no change, reduced optic flow, and reduced distal cues) and participant group (young vs. healthy elder participants vs. MCI patients), followed by post hoc multiple comparisons with Bonferroni correction and statistical power analysis.[31] There were no significant interactions (all F(4,277) < 2.25, and all p > 0.064) or main effects of environmental conditions (all F(2,277) < 1.14, and all p > 0.320) on any parameters. However, MCI patients showed larger angular encoding gain than healthy older participants (i.e., g_2; F(2,277) = 7.44, p = 0.037, power = 94.7% at alpha level 0.05 and effect size 0.232, Figure 4B) and significant gain overall (i.e., $g_2 > 1$; two-tailed t test with t(36) = 4.65, p < 0.001).

MCI patients also showed more noisy generation of inbound distances (i.e., greater σ) than healthy elderly (F(2,277) = 6.86, p < 0.001, power = 92.8% at alpha level 0.05 and effect size 0.223, Figure 4F) and of inbound direction (i.e., greater ν, F(2,277) = 22.6, p < 0.001, power = 99.9% at alpha level 0.05 and effect size 0.404, Figure 4G). Although there was no significant effect of group on speed gain k (F(2,277) = 0.75, p = 1.000, Figure 4A), both healthy elderly and MCI patients showed speed gain (i.e., $k > 1$; two-tailed t test with t(32) = 6.25, p < 0.001 for healthy elderly, and t(36) = 3.58, p = 0.001 for MCI patients). Similarly, there was no significant effect of group on distance forgetting or leak β (F(2,277) = 1.02, p = 0.677, Figure 4C); however, MCI patients had significant forgetting of distance (two-tailed t test on β with t(38) = 2.89, p = 0.006) but not young and healthy elderly participants. Finally, there were group differences in angular production error g_3 (F(2,277) = 0.58, p = 1.000, Figure 4D), but all three groups show significant regression-to-the-mean effects on the inbound angle (i.e., $g_3 < 1$; two-tailed t test with t(23) = −3.51, p = 0.002 on young participants; t(32) = −3.38, p = 0.002 on healthy elderly; t(36) = −5.95, p < 0.001 on MCI patients) but not the inbound distance (i.e., $m_3 < 1$; two-tailed t test have all p > 0.636).

Angular PI is impaired prior to Alzheimer's dementia

To investigate the effect of AD pathology, we carried out a two-way ANOVA on the MCI patients with CSF status available for all GLAMPI parameters, with the factors environmental condition and CSF status. We found an interaction between environmental conditions and participant group in angular production g_3 (p = 0.012), where MCI+ patients show significant regression to the mean when producing the intended inbound angle under reduced distal cue conditions compared with no-change conditions (Figure 5H). Such interaction was not observed in MCI− patients. We also found a borderline effect of environmental condition on distance production m_3 (F(2,62) = 2.94, p = 0.060), where both MCI+ and MCI− patients show significant regression-to-the-mean effect when producing the inbound distance under reduced optic flow conditions (Figure 5I), perhaps reflecting uncertainty in the distance to walk in the current trial due to reduced optic flow.

CellPress
OPEN ACCESS

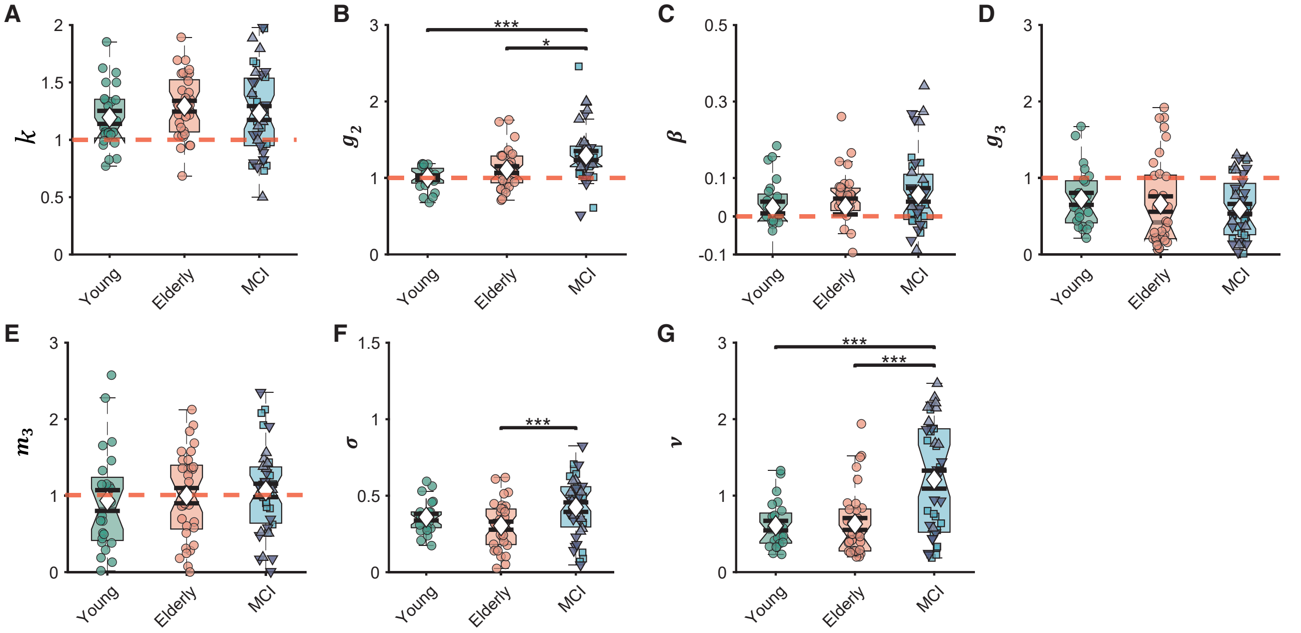

Figure 4. GLAMPI parameters across groups

Showing young (green), healthy elderly (orange) participants, and MCI patients (blue).

(A–G) k,speed gain; g_2, angular gain; β, leak term; g_3, angular production slope; m_3, distance production slope; σ, distance noise amplitude; and ν, angular noise amplitude. Up-pointing triangles represent MCI+, down-pointing triangles represent MCI−, and squares represent MCI unknown. Each dot represents a parameter estimated from the data of one participant (averaged across three conditions given the absence of an effect of condition or interaction). Boxplots are shown as in Figure 1. Red dashed lines (reference lines) mark the optimal parameter value (i.e., no error occurs at this value). *$p < 0.05$, **$p < 0.01$, and ***$p < 0.001$ are marked from the two-way ANOVA with post hoc multiple comparisons with Bonferroni correction. See also Figure S4 for deviations of parameter estimates from their optimal values for healthy elderly and Figure S5 for separated MCI groups.

Main effects of group revealed a larger angular encoding gain in MCI+ than MCI− patients (i.e., g_2: $F(1,62) = 6.87$, $p = 0.011$, power = 77.1% at alpha level 0.05 and effect size 0.333, Figure 5B), but no significant difference in speed gain (i.e., k: $F(1,62) = 0.37$, $p = 0.546$, Figure 5A). In addition, MCI+ patients show greater noise in generating the inbound direction than MCI− patients (i.e., ν: $F(1,62) = 22.2$, $p < 0.001$, power = 99.8% at alpha level 0.05 and effect size 0.598, Figure 5G) but not in generating the inbound distance (i.e., σ: $F(1,62) = 0.520$, $p = 0.473$, Figure 5F). We found no significant difference between the two groups in distance forgetting (i.e., β: $F(1,62) = 0.53$, $p = 0.468$, Figure 5C) or distance production error (i.e., m_3: $F(1,62) = 0.16$, $p = 0.695$, Figure 5E). Finally, we looked in more detail at the increased angular gain (g_2) in the pooled MCI group compared with healthy elderly by running a two-way ANOVA with all groups separately, finding that it was mainly driven by MCI+ patients, whereas MCI− patients showed no difference (MCI− vs. healthy elderly, $F(4, 271) = 6.88$, $p = 1.00$; MCI+ vs. healthy elderly $F(4, 271) = 6.88$, $p < 0.001$; Figure S5).

Classification of AD within MCI based on GLAMPI parameters

We assessed the ability to classify the MCI group from healthy elderly participants and to classify CSF status within MCI patients by using GLAMPI parameters or direct measures of performance using the receiver operator characteristic (ROC) (Figure 6; see STAR Methods). For classifying MCI vs. healthy elderly, the proportional angular error was reliably discriminative but not significantly better than the GLAMPI angular noise parameter (DeLong's test, $p = 0.223$ with var(1) = 0.004, var(2) = 0.003, and cov = 0.001; see Figures 6A and 6B).

For classifying MCI+ vs. MCI−, the GLAMPI parameters for encoding angular gain (g_2) and angular noise (ν) were strongly discriminative, uniquely among GLAMPI parameters (Figure 6D; DeLong's test on the corresponding ROC curves: g_2 vs. all other parameters: $p < 0.001$ with var(1) = 0.013, var(2) < 0.005, $-4.0 \times 10^{-4} < \text{cov} \leq -1.0 \times 10^{-4}$; ν vs. all the other parameters: $p < 0.001$ with var(1) = 0.014, var(2) < 0.005, $0 < \text{cov} \leq 6.0 \times 10^{-4}$), although these were not significantly better than the proportional angular or linear errors (Figure 6C; DeLong's tests on the corresponding ROC curves of ν and the proportional angular error with $p = 0.892$ with var(1) = 0.014, var(2) = 0.013, and cov = −0.002).

To conclude, the GLAMPI model indicates that the impaired behavioral measures of PI in MCI+ vs. MCI− reflect increased angular gain and angular variance. We could not make such an inference from the behavioral measures alone, although they are equally diagnostic of MCI+ because both the return distances and angles show equal impairment.

DISCUSSION

Impaired PI is a promising cognitive biomarker of early AD and likely reflects dysfunction in brain regions such as entorhinal

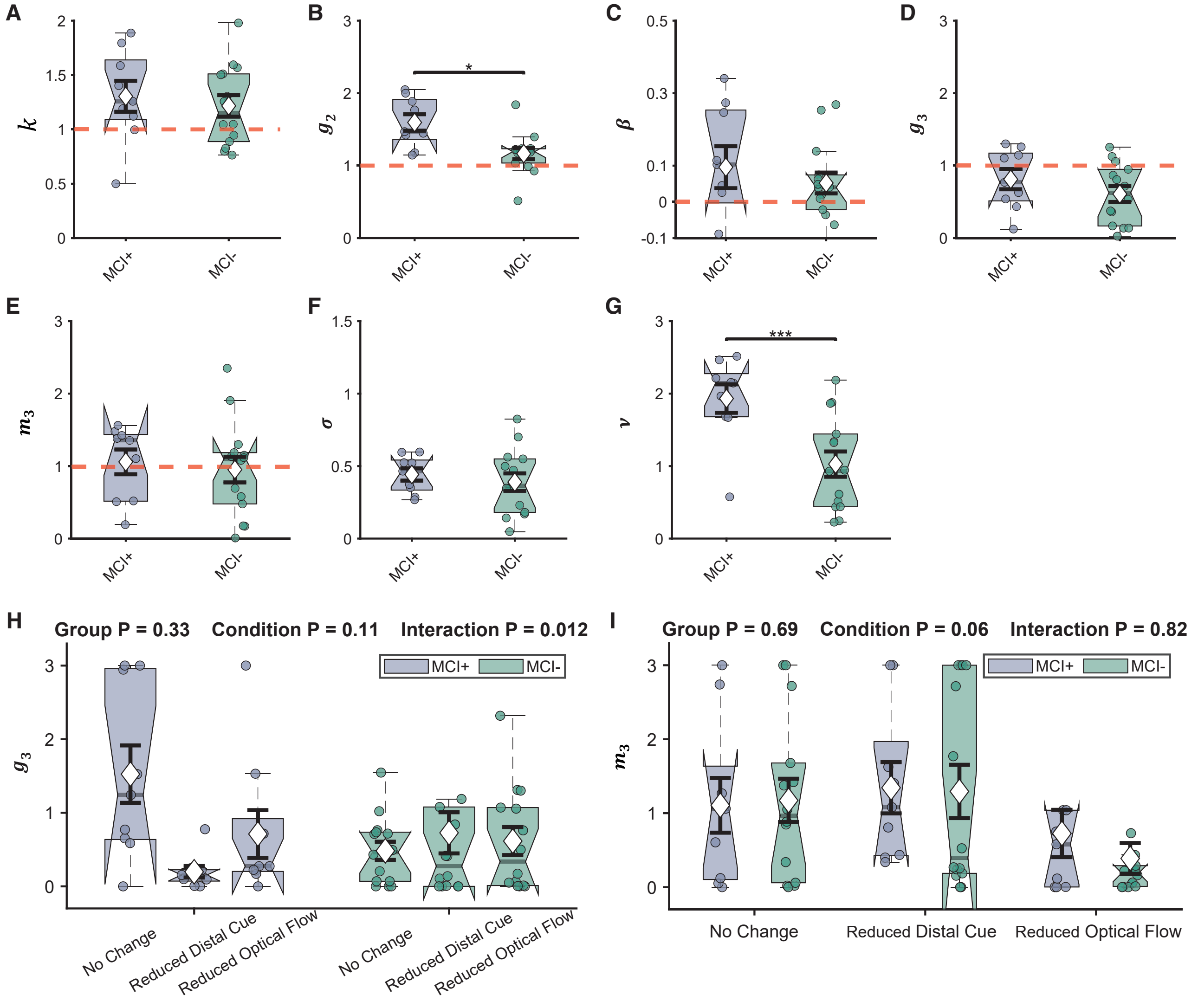

Figure 5. GLAMPI parameter comparisons between MCI+ and MCI– patients, and conditions

(A–G) Estimated parameters (k, speed gain; g_2, angular gain; β, leak term; g_3, angular production slope; m_3, distance production slope; σ, distance noise amplitude; and ν, angular noise amplitude) were averaged across conditions for each participant. Each dot represents a parameter estimated from the data of one participant (averaged across three conditions). Red dashed lines (reference lines) mark the optimal parameter value (i.e., no error occurs at this value). *$p < 0.05$, **$p < 0.01$, and ***$p < 0.001$.

(H) The interaction effect of g_3, where MCI+ patients show significantly larger g_3 in the no-change condition than that in the reduced distal cue condition.

(I) Borderline condition effect of m_3, where MCI+ and MCI− patients show smaller m_3 in the reduced optic flow condition than the other two conditions. Boxplots are shown as in Figure 1.

cortex, which manifest neuropathology early in AD.[1,2] To identify the mechanisms underlying this deficit, we developed a GLAMPI (Figure 2) to explain the trial-by-trial performance of inbound paths in triangle completion tasks.

We modeled the contributions of linear and angular PI separately, motivated by the different vestibular origins of linear and angular motion signals,[20] the separate neural processing of speed[21,22] and angular velocity[23–25] in the brain, and the uncertainty in how these inputs are combined (the directional modulation of medial entorhinal cell firing[29] reflects head direction rather than movement direction, which is insufficient for PI[30]). Our approach offers additional sensitivity to different potential sources of errors related to aging and AD, extending previous models,[11] with relevance for associations between vestibular and cognitive deficits in AD.[32]

Separate consideration of the linear distance and angular turn comprising the inbound path (Figure 1) shows that MCI can be distinguished from healthy elderly in both the distance and direction walked, with an aging effect also present in the proportional distance walked by younger and older participants. The pooled MCI participants comprise a heterogeneous group, including a variety of non-AD causes of cognitive impairment. However, analyzing the MCI patients with CSF biomarkers indicative of underlying AD (MCI+ vs. MCI−) revealed that

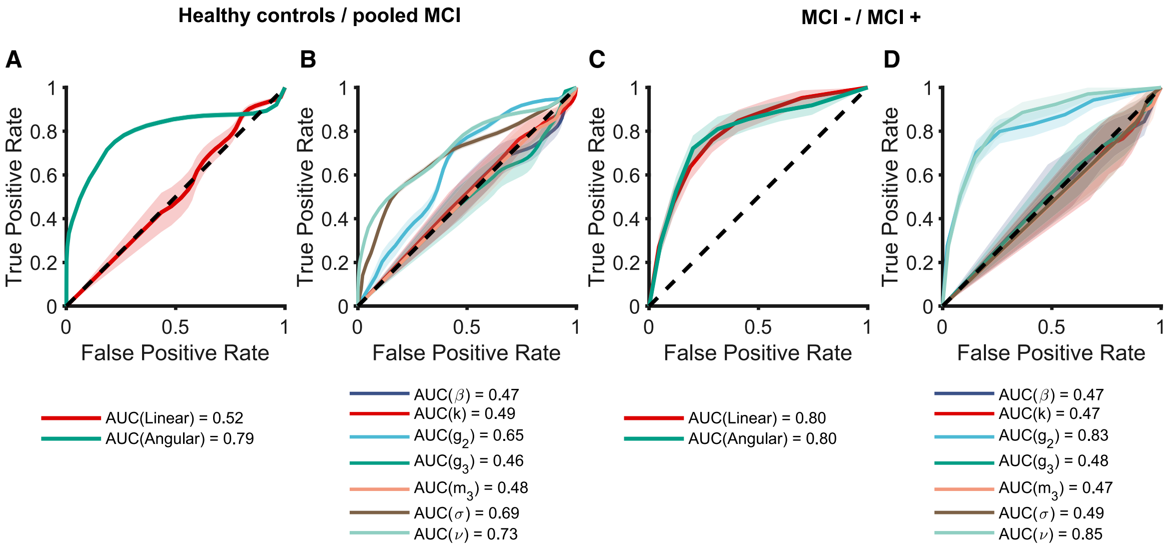

Figure 6. Classification of participant group using behavioral performance and GLAMPI parameters

Showing receiver operating characteristic (ROC) curves and associated areas under the curve (AUC). A support vector machine was used to assess the ability of the path integration performance (A and C) and the fitted GLAMPI parameters (B and D) to classify MCI status in elderly participants (A and B), and to classify the CSF status in the MCI patients (C and D). A linear kernel was adopted for the classification. Mean ROC curves are cross-validated using 1,000 repetitions where, in each repetition, the test set was obtained from a hold-out of 40% of the total sample. Mean AUC values are reported in the legends. Support vector machines trained used the proportional linear error (red) or proportional angular error (green) in the behavioral data or the fitted GLAMPI parameters for each participant. Shaded areas indicate the SEM. Dashed black line indicates the chance level.

MCI+ patients under-walked and over turned more than MCI− (Figure 1). Although these behavioral results support the known overall deficit in PI in MCI with AD biomarkers,[2] they could reflect a variety of underlying cognitive issues, such as challenges in spatial perception, integration of spatial cues, and potential biases in direction or distance estimation. The aim of our modeling was to understand the pattern of errors in terms of more fundamental processes underlying the generation of behavioral responses.

Our model follows the literature indicating three sources of error in PI: (1) encoding the outbound movement,[11–13] (2) calculation of the intended inbound path, including integration of velocity along the outbound path,[14,15] and (3) production of the intended inbound path.[16,17,33,34] A minimal parameterization of the resulting generative model (GLAMPI) was found by fitting the data of healthy elderly participants while minimizing model complexity using the AIC (Figure 2), bearing in mind that patients might complete only small numbers of trials. By fitting the GLAMPI parameters to the pattern of return paths across several trials for each condition, we interpret the raw behavioral responses into potential underlying mechanisms. Each parameter was necessary to improve the fit (Figure 2C), supporting the presence of all the previously suggested types of error and together providing the best explanation for the data consistent with our model of encoding, calculation, and production errors. For example, leaky integration is described as a calculation error because it continues to affect the representation of the first outbound leg until the calculation of the intended return path, following the observation of Harootonian et al.[13]

Applying the GLAMPI to our groups, we found that differences in performance between healthy elderly and pooled MCI were captured by increased gain in encoding the rotation between the legs of the outbound path and by increased noise terms, which reflect unmodeled variance in the inbound path, with the increase in angular noise particularly noticeable (Figure 4). When the MCI group was subdivided according to the CSF biomarkers of AD (MCI+ or MCI−), the difference in performance between MCI+ and MCI− patients was reflected only in the angular components of the model (Figure 5), namely ncreased gain in encoding the rotation between the legs of the outbound path and increased variability in the direction of the inbound path. Given that MCI diagnosis without access to biomarkers is a more common clinical presentation, comparing MCI+ and MCI− alongside the contrast between pooled MCI and healthy elderly highlights the importance of the angular results given that the increased linear variance observed between pooled MCI and healthy aging is not diagnostic of incipient AD. Interestingly, over-turning during the outbound paths is specific to the MCI+ group, with the MCI− and MCI without biomarkers groups not showing significant divergence from healthy elderly (Figure S5). Thus, our model pinpoints a specific cause for the observed behavioral impairment in AD—a mental over-turning during the outbound path—in addition to the increased variance in return direction. This finding holds important implications for future development of more targeted tasks aimed at identifying the cognitive impairments in early AD.

Our analysis revealed an interaction between environmental conditions and MCI subgroups on the regression to the mean of the produced return direction (g_3). Specifically, reducing the distal visual orientation cues during the inbound part of the trial produced more stereotypical angular responses (i.e., less sensitivity to the correct homing direction on each trial) in the MCI+ group compared with the no-change environmental condition. This pattern corroborates the overall results, namely, that the

MCI+ cohort are impaired at integrating angular self-motion information and therefore are more reliant on visual orientation cues. Although not achieving statistical significance, we also observed a trend in which both MCI+ and MCI− exhibited stronger regression-to-the-mean produced return distance when optic flow cues were reduced, suggesting a weakness in judging walked distance that is exacerbated by reduced optic flow but is not specific to AD.

The angular parameters (g_2 and ν) are unique among the GLAMPI model components in providing a good basis for classification according to biomarker status (Figure 6). This contrasts with the (equally diagnostic) behavioral measures, in which both angular and distance measures are equally impaired. The parameter g_2 is also more specific in separating only the MCI+ group from the healthy elderly group (i.e., g_2 does not differ between MCI− and healthy elderly, Figure S5), whereas behavioral over-turning relative to healthy elderly was seen in all MCI subgroups (albeit most strongly in MCI+, Figure 1F). Thus, the model allows a more specific interpretation of the deficit in MCI+ than would be possible without such a model. The increased gain (g_2) is not the only possible explanation of increased over-turning in the MCI+ group, e.g., leaky integration of distance shortening the first leg of the outbound path more than the second leg would be another (although in our data, parameter β was not different from zero). In addition, the increased g_2 also explains the under-walking seen in this group (because they think they are closer to the start location when they commence the inbound path). Given the importance of g_2 in our results, it is worth noting that head posture (specifically pitch) could potentially influence the vestibular encoding of rotation;[35] however, we did not observe any differences between groups in head pitch during the outbound path (Figure S1F).

Our previous report on PI within the MCI population emphasized the difference in absolute distance error between the start and return locations in MCI+ vs. MCI−[2], which was more discriminative than either the proportional distance error or proportional angular error alone. The use of GLAMPI allows the behavioral data to be interpreted in terms of increased angular gain and angular variance in the MCI+ group, providing a specific interpretation for the behavioral impairments reflected in absolute distance errors, over-turning and under-walking. There are other differences between the two studies reflecting our current focus on characterizing PI mechanisms. First, we required participants to show a clear indication of movement direction by walking at least 0.5 m before pressing the button to indicate that they had finished. Exceptions to this tended to occur at the start of testing and more so in MCI+ than MCI− group (10.1% vs. 2.4% of trials, see STAR Methods). Second, we excluded participants from conditions in which they completed fewer than seven trials (out of the nine available), due to the need to estimate 7 GLAMPI parameters. Third, we analyzed the direction of return paths that went out of bounds (29.7% for MCI− and 29.3% for MCI+, see STAR Methods), only discarding the distance information rather than the whole trial. Finally, technical data were lost from two participants (MCI+, MCI unknown). The inclusion of very short return distances (<0.5 m), which may reflect general misunderstanding rather than a specific PI impairment, and the exclusion of the directions of out-of-bounds trials (which tend to have large angular error) will have enhanced the diagnostic value of distance errors compared with angular errors in the previous study.

The comparison of healthy younger and healthy older participants did not reveal significant differences in model parameters, suggesting that the differences associated with AD biomarker status are not a simple extension of effects of healthy aging[11,19,36–39] but instead an effect specific to AD pathology. Consistent with this, there was no effect of over-turning in the healthy elderly (see Figure 1). However, the behavioral results do show greater under-walking for older vs. younger participants and a higher proportion of out-of-bounds trials (Figure S1). The model interprets the under-walking as a non-significantly greater leaky integration of distance, combined with the longer duration of outbound paths taken by older participants (as younger participants were tested in a smaller room, see STAR Methods and Figures S1C and S6). Thus, the model partly credits the additional under-walking to a difference in testing conditions rather than in underlying processes. Broadly similar performance of older and younger participants on triangle completion is consistent with previous studies involving actual walking[37,40] (as opposed to desktop VR[19,37] or use of a wheelchair[37,40]). In line with our observations from Figure S1C, the reduced size of the triangles experienced by the young participants likely contributed to their significantly fewer instances of out-of-bound trials when contrasted with the elderly group (Figure S1B). However, it is important to note that this should not impact the proportional distance error (Figure 1C), given that out-of-bound trials were only used for computing angular measures.

The impairment in angular PI observed in MCI+ patients suggests a deficit in integrating angular velocity to update spatial representations. The vestibular nuclei signal angular acceleration detected by the semi-circular canals[20] which is integrated over time into angular head velocity, possibly in the nucleus prepositus[41] and dorsal tegmental nucleus.[42,43] This angular head velocity is a prerequisite of the head-direction signal, found along the circuit from the lateral mamillary nuclei, anterior-dorsal thalamic nucleus, retrosplenial cortex, and presubiculum and entorhinal cortex.[44] Alternatively to the head-direction circuit, self-motion information also reaches entorhinal cortex from the medial septum[22,45,46] and posterior parietal cortex.[47] Human neuroimaging relying on optic flow self-motion also proposes roles for retrosplenial cortex[48] and cerebellum[49] in processing heading rotation, with retrosplenial hypometabolism observed in MCI.[50] Therefore, the site for our observed deficit could well be entorhinal cortex, given its role in PI[27,28] and association with AD neuropathology,[7,51] or the pathways bringing angular velocity to it. Entorhinal grid cells are thought to update self-location on the basis of self-motion, while interfacing with the more sensory-driven place cell representation in hippocampus.[52] However, it is not clear how directional and linear information are combined, given that the directional modulation of medial entorhinal cell firing[29] reflects head direction rather than movement direction and so is insufficient for PI,[30] whereas an allocentric movement signal has not yet been identified in mammals (unlike *Drosophila*[53,54]). Future investigation might address the precise etiology of this impairment and its relationship to other aspects of cognition impaired early in AD.[55,56]

This study comes with limitations. The number of participants with proven biomarker status was relatively small. Post hoc statistical power analysis revealed there was 77% chance of detecting the observed difference in angular gain g_2 between MCI+ and MCI−, which we consider as moderate, whereas the power to detect all other significant effects between the groups was above 90%, which we consider strong, with desired power typically 80% or more (see results for details). Any conclusions drawn should be treated with caution until further validation with a larger study. The pooled MCI group is heterogeneous, encompassing a variety of non-AD causes of cognitive impairment, further complicating the extrapolation of those results to other MCI populations. Finally, although the GLAMPI presents a comprehensive tool for evaluating sources of PI, it does not necessarily encompass every potential source of error and might be further refined in future.

In conclusion, our computational model can effectively capture and distinguish different types of errors associated with PI deficits specific to AD and highlights the angular components. As such, these results can be used to refine the design of spatial navigation tests for detection of prodromal AD and facilitate translational research aimed at identifying the association between AD pathology and the emergence of behavioral deficits around dementia onset.

STAR★METHODS

Detailed methods are provided in the online version of this paper and include the following:

- KEY RESOURCES TABLE
- RESOURCE AVAILABILITY
  - Lead contact
  - Materials availability
  - Data and code availability
- EXPERIMENTAL MODEL AND STUDY PARTICIPANT DETAILS
- METHOD DETAILS
  - Path Integration task
  - Data pre-processing
  - Calculation of path integration error
  - The computational model
  - Outbound path additional information
  - Deriving the distance encoding component from a leaky-integrator model
  - Relationship with the leaky-integrator model used in desktop VR
  - Correlation between model parameters and age or educational duration
- QUANTIFICATION AND STATISTICAL ANALYSIS

SUPPLEMENTAL INFORMATION

Supplemental information can be found online at https://doi.org/10.1016/j.cub.2023.09.047.

ACKNOWLEDGMENTS

We thank David Howett for providing the data from patients and elderly controls and Talfan Evans for early discussion of the work. We would like to thank Sara De Felice for providing the illustration for Figure S1A. We would like to thank Caswell Barry for providing feedback on the manuscript and Misun Kim for proofreading. This work was support by the Wellcome Principal Research Fellowship (222457/Z/21/Z) awarded to N.B., China International Postdoctoral Exchange Fellowship Program (No. PC2021005) awarded to Z.J., and the Biotechnology and Biological Sciences Research Council (grant number BB/T008709/1) awarded to K.R.

AUTHOR CONTRIBUTIONS

A.C. Z.J., and N.B. conceptualized and designed the research. A.C. and N.B. developed the initial version of the model. Z.J. and N.B. developed the current version of the model. A.C. programmed the virtual reality path integration task. A.C. pre-processed the data. A.C. and Z.J. analyzed the data. A.C., Z.J., K.R., and N.B. interpreted the results. All authors drafted the paper. N.B. supervised the work.

DECLARATION OF INTERESTS

The authors declare no competing interests.

INCLUSION AND DIVERSITY

We support inclusive, diverse, and equitable conduct of research.

Received: June 9, 2023
Revised: August 21, 2023
Accepted: September 20, 2023
Published: October 11, 2023

REFERENCES

1. Mokrisova, I., Laczo, J., Andel, R., Gazova, I., Vyhnalek, M., Nedelska, Z., Levcik, D., Cerman, J., Vlcek, K., and Hort, J. (2016). Real-space path integration is impaired in Alzheimer's disease and mild cognitive impairment. Behav. Brain Res. *307*, 150–158. https://doi.org/10.1016/j.bbr.2016.03.052.
2. Howett, D., Castegnaro, A., Krzywicka, K., Hagman, J., Marchment, D., Henson, R., Rio, M., King, J.A., Burgess, N., and Chan, D. (2019). Differentiation of mild cognitive impairment using an entorhinal cortex-based test of virtual reality navigation. Brain *142*, 1751–1766. https://doi.org/10.1093/brain/awz116.
3. Burgess, N. (2008). Spatial cognition and the brain. Ann. N. Y. Acad. Sci. *1124*, 77–97. https://doi.org/10.1196/annals.1440.002.
4. McNaughton, B.L., Battaglia, F.P., Jensen, O., Moser, E.I., and Moser, M.B. (2006). Path integration and the neural basis of the 'cognitive map'. Nat. Rev. Neurosci. *7*, 663–678. https://doi.org/10.1038/nrn1932.
5. Hafting, T., Fyhn, M., Molden, S., Moser, M.B., and Moser, E.I. (2005). Microstructure of a spatial map in the entorhinal cortex. Nature *436*, 801–806. https://doi.org/10.1038/nature03721.
6. Jacobs, J., Weidemann, C.T., Miller, J.F., Solway, A., Burke, J.F., Wei, X.X., Suthana, N., Sperling, M.R., Sharan, A.D., Fried, I., and Kahana, M.J. (2013). Direct recordings of grid-like neuronal activity in human spatial navigation. Nat. Neurosci. *16*, 1188–1190. https://doi.org/10.1038/nn.3466.
7. Braak, H., and Braak, E. (1991). Neuropathological stageing of Alzheimer-related changes. Acta Neuropathol. *82*, 239–259. https://doi.org/10.1007/BF00308809.
8. Bierbrauer, A., Kunz, L., Gomes, C.A., Luhmann, M., Deuker, L., Getzmann, S., Wascher, E., Gajewski, P.D., Hengstler, J.G., Fernandez-Alvarez, M., et al. (2020). Unmasking selective path integration deficits in Alzheimer's disease risk carriers. Sci. Adv. *6*, eaba1394. https://doi.org/10.1126/sciadv.aba1394.
9. Kunz, L., Schröder, T.N., Lee, H., Montag, C., Lachmann, B., Sariyska, R., Reuter, M., Stirnberg, R., Stöcker, T., Messing-Floeter, P.C., et al. (2015). Reduced grid-cell-like representations in adults at genetic risk for

Alzheimer's disease. Science *350*, 430–433. https://doi.org/10.1126/science.aac8128.

10. Segen, V., Ying, J., Morgan, E., Brandon, M., and Wolbers, T. (2022). Path integration in normal aging and Alzheimer's disease. Trends Cogn. Sci. *26*, 142–158. https://doi.org/10.1016/J.TICS.2021.11.001.
11. Stangl, M., Kanitscheider, I., Riemer, M., Fiete, I., and Wolbers, T. (2020). Sources of path integration error in young and aging humans. Nat. Commun. *11*, 2626. https://doi.org/10.1038/s41467-020-15805-9.
12. Fujita, N., Klatzky, R.L., Loomis, J.M., and Golledge, R.G. (1993). The encoding-error model of pathway completion without vision. Geogr. Anal. *25*, 295–314. https://doi.org/10.1111/J.1538-4632.1993.TB00300.X.
13. Harootonian, S.K., Wilson, R.C., Hejtmánek, L., Ziskin, E.M., and Ekstrom, A.D. (2020). Path integration in large-scale space and with novel geometries: comparing vector addition and encoding-error models. PLoS Comput. Biol. *16*, e1007489. https://doi.org/10.1371/JOURNAL.PCBI.1007489.
14. Lappe, M., Jenkin, M., and Harris, L.R. (2007). Travel distance estimation from visual motion by leaky path integration. Exp. Brain Res. *180*, 35–48. https://doi.org/10.1007/s00221-006-0835-6.
15. Lappe, M., Stiels, M., Frenz, H., and Loomis, J.M. (2011). Keeping track of the distance from home by leaky integration along veering paths. Exp. Brain Res. *212*, 81–89. https://doi.org/10.1007/s00221-011-2696-x.
16. Chrastil, E.R., and Warren, W.H. (2021). Executing the homebound path is a major source of error in homing by path integration. J. Exp. Psychol. Hum. Percept. Perform. *47*, 13–35. https://doi.org/10.1037/XHP0000875.
17. Chrastil, E.R., and Warren, W.H. (2017). Rotational error in path integration: encoding and execution errors in angle reproduction. Exp. Brain Res. *235*, 1885–1897. https://doi.org/10.1007/s00221-017-4910-y.
18. Petzschner, F.H., and Glasauer, S. (2011). Iterative bayesian estimation as an explanation for range and regression effects: a study on human path integration. J. Neurosci. *31*, 17220–17229. https://doi.org/10.1523/JNEUROSCI.2028-11.2011.
19. Harris, M.A., and Wolbers, T. (2012). Ageing effects on path integration and landmark navigation. Hippocampus *22*, 1770–1780. https://doi.org/10.1002/HIPO.22011.
20. Carriot, J., Jamali, M., Brooks, J.X., and Cullen, K.E. (2015). Integration of canal and otolith inputs by central vestibular neurons is subadditive for both active and passive self-motion: implication for perception. J. Neurosci. *35*, 3555–3565. https://doi.org/10.1523/JNEUROSCI.3540-14.2015.
21. Kropff, E., Carmichael, J.E., Moser, M.B., and Moser, E.I. (2015). Speed cells in the medial entorhinal cortex. Nature *523*, 419–424. https://doi.org/10.1038/nature14622.
22. Fuhrmann, F., Justus, D., Sosulina, L., Kaneko, H., Beutel, T., Friedrichs, D., Schoch, S., Schwarz, M.K., Fuhrmann, M., and Remy, S. (2015). Locomotion, theta oscillations, and the speed-correlated firing of hippocampal neurons are controlled by a medial septal glutamatergic circuit. Neuron *86*, 1253–1264. https://doi.org/10.1016/J.NEURON.2015.05.001.
23. Spalla, D., Treves, A., and Boccara, C.N. (2022). Angular and linear speed cells in the parahippocampal circuits. Nat. Commun. *13*, 1907. https://doi.org/10.1038/s41467-022-29583-z.
24. Stackman, R.W., and Taube, J.S. (1998). Firing properties of rat lateral mammillary single units: head direction, head pitch, and angular head velocity. J. Neurosci. *18*, 9020–9037. https://doi.org/10.1523/JNEUROSCI.18-21-09020.1998.
25. O'Mara, S.M., Rolls, E.T., Berthoz, A., and Kesner, R.P. (1994). Neurons responding to whole-body motion in the primate hippocampus. J. Neurosci. *14*, 6511–6523. https://doi.org/10.1523/JNEUROSCI.14-11-06511.1994.
26. Burak, Y., and Fiete, I.R. (2009). Accurate path integration in continuous attractor network models of grid cells. PLoS Comput. Biol. *5*, e1000291. https://doi.org/10.1371/JOURNAL.PCBI.1000291.
27. Gil, M., Ancau, M., Schlesiger, M.I., Neitz, A., Allen, K., De Marco, R.J., and Monyer, H. (2017). Impaired path integration in mice with disrupted grid cell firing. Nat. Neurosci. *21*, 81–91. https://doi.org/10.1038/s41593-017-0039-3.
28. Tennant, S.A., Fischer, L., Garden, D.L.F., Gerlei, K.Z., Martinez-Gonzalez, C., McClure, C., Wood, E.R., and Nolan, M.F. (2018). Stellate cells in the medial entorhinal cortex are required for spatial learning. Cell Rep. *22*, 1313–1324. https://doi.org/10.1016/j.celrep.2018.01.005.
29. Sargolini, F., Fyhn, M., Hafting, T., McNaughton, B.L., Witter, M.P., Moser, M.B., and Moser, E.I. (2006). Conjunctive representation of position, direction, and velocity in entorhinal cortex. Science *312*, 758–762. https://doi.org/10.1126/science.1125572.
30. Raudies, F., Brandon, M.P., Chapman, G.W., and Hasselmo, M.E. (2015). Head direction is coded more strongly than movement direction in a population of entorhinal neurons. Brain Res. *1621*, 355–367. https://doi.org/10.1016/J.BRAINRES.2014.10.053.
31. Faul, F., Erdfelder, E., Lang, A.G., and Buchner, A. (2007). G*Power 3: a flexible statistical power analysis program for the social, behavioral, and biomedical sciences. Behav. Res. Methods *39*, 175–191. https://doi.org/10.3758/bf03193146.
32. Coughlan, G., Plumb, W., Zhukovsky, P., Aung, M.H., and Hornberger, M. (2023). Vestibular contribution to path integration deficits in 'at-genetic-risk' for Alzheimer's disease. PLoS One *18*, e0278239. https://doi.org/10.1371/JOURNAL.PONE.0278239.
33. Israël, I., Bronstein, A.M., Kanayama, R., Faldon, M., and Gresty, M.A. (1996). Visual and vestibular factors influencing vestibular "navigation.". Exp. Brain Res. *112*, 411–419. https://doi.org/10.1007/BF00227947.
34. Becker, W., Jürgens, R., and Boss, T. (2000). Vestibular perception of self-rotation in different postures: a comparison between sitting and standing subjects. Exp. Brain Res. *131*, 468–476. https://doi.org/10.1007/s002219900321.
35. Choi, J.Y., Koo, Y.J., Song, J.M., Kim, H.J., and Kim, J.S. (2023). Effect of a false inertial cue in the velocity-storage circuit on head posture and inertia perception. J. Neurosci. *43*, 1530–1539. https://doi.org/10.1523/JNEUROSCI.1148-22.2023.
36. Bates, S.L., and Wolbers, T. (2014). How cognitive aging affects multisensory integration of navigational cues. Neurobiol. Aging *35*, 2761–2769. https://doi.org/10.1016/J.NEUROBIOLAGING.2014.04.003.
37. Adamo, D.E., Briceño, E.M., Sindone, J.A., Alexander, N.B., and Moffat, S.D. (2012). Age differences in virtual environment and real world path integration. Front. Aging Neurosci. *4*, 26. https://doi.org/10.3389/fnagi.2012.00026.
38. Bécu, M., Sheynikhovich, D., Tatur, G., Agathos, C.P., Bologna, L.L., Sahel, J.A., and Arleo, A. (2020). Age-related preference for geometric spatial cues during real-world navigation. Nat. Hum. Behav. *4*, 88–99. https://doi.org/10.1038/s41562-019-0718-z.
39. Tansan, M., Nguyen, K.V., and Newcombe, N.S. (2022). Spatial navigation in childhood and aging. Annu. Rev. Dev. Psychol. *4*, 253–272. https://doi.org/10.1146/annurev-devpsych-121020-031846.
40. Allen, G.L., Kirasic, K.C., Rashotte, M.A., and Haun, D.B.M. (2004). Aging and path integration skill: kinesthetic and vestibular contributions to wayfinding. Percept. Psychophys. *66*, 170–179. https://doi.org/10.3758/BF03194870.
41. Butler, W.N., and Taube, J.S. (2015). The nucleus prepositus hypoglossi contributes to head direction cell stability in rats. J. Neurosci. *35*, 2547–2558. https://doi.org/10.1523/JNEUROSCI.3254-14.2015.
42. Bassett, J.P., and Taube, J.S. (2001). Neural correlates for angular head velocity in the rat dorsal tegmental nucleus. J. Neurosci. *21*, 5740–5751. https://doi.org/10.1523/JNEUROSCI.21-15-05740.2001.
43. Sharp, P.E., Tinkelman, A., and Cho, J. (2001). Angular velocity and head direction signals recorded from the dorsal tegmental nucleus of gudden in the rat: implications for path integration in the head direction cell circuit. Behav. Neurosci. *115*, 571–588. https://doi.org/10.1037/0735-7044.115.3.571.

CellPress
OPEN ACCESS

44. Taube, J.S. (2007). The head direction signal: origins and sensory-motor integration. Annu. Rev. Neurosci. *30*, 181–207. https://doi.org/10.1146/annurev.neuro.29.051605.112854.

45. Brandon, M.P., Bogaard, A.R., Libby, C.P., Connerney, M.A., Gupta, K., and Hasselmo, M.E. (2011). Reduction of theta rhythm dissociates grid cell spatial periodicity from directional tuning. Science *332*, 595–599. https://doi.org/10.1126/science.1201652.

46. Koenig, J., Linder, A.N., Leutgeb, J.K., and Leutgeb, S. (2011). The spatial periodicity of grid cells is not sustained during reduced theta oscillations. Science *332*, 592–595. https://doi.org/10.1126/SCIENCE.1201685.

47. Whitlock, J.R., Sutherland, R.J., Witter, M.P., Moser, M.B., and Moser, E.I. (2008). Navigating from hippocampus to parietal cortex. Proc. Natl. Acad. Sci. USA *105*, 14755–14762. https://doi.org/10.1073/PNAS.0804216105.

48. Chrastil, E.R., Sherrill, K.R., Hasselmo, M.E., and Stern, C.E. (2016). Which way and how far? Tracking of translation and rotation information for human path integration. Hum. Brain Mapp. *37*, 3636–3655. https://doi.org/10.1002/HBM.23265.

49. Chrastil, E.R., Sherrill, K.R., Aselcioglu, I., Hasselmo, M.E., and Stern, C.E. (2017). Individual differences in human path integration abilities correlate with gray matter volume in retrosplenial cortex, hippocampus, and medial prefrontal cortex. eNeuro *4*, 346–362. https://doi.org/10.1523/ENEURO.0346-16.2017.

50. Nestor, P.J., Fryer, T.D., Ikeda, M., and Hodges, J.R. (2003). Retrosplenial cortex (BA 29/30) hypometabolism in mild cognitive impairment (prodromal Alzheimer's disease). Eur. J. Neurosci. *18*, 2663–2667. https://doi.org/10.1046/J.1460-9568.2003.02999.X.

51. Lopera, F., Marino, C., Chandrahas, A.S., O'Hare, M., Villalba-Moreno, N.D., Aguillon, D., Baena, A., Sanchez, J.S., Vila-Castelar, C., Ramirez Gomez, L., et al. (2023). Resilience to autosomal dominant Alzheimer's disease in a Reelin-COLBOS heterozygous man. Nat. Med. *29*, 1243–1252. https://doi.org/10.1038/s41591-023-02318-3.

52. Chen, G., Lu, Y., King, J.A., Cacucci, F., and Burgess, N. (2019). Differential influences of environment and self-motion on place and grid cell firing. Nat. Commun. *10*, 630. https://doi.org/10.1038/s41467-019-08550-1.

53. Lu, J., Behbahani, A.H., Hamburg, L., Westeinde, E.A., Dawson, P.M., Lyu, C., Maimon, G., Dickinson, M.H., Druckmann, S., and Wilson, R.I. (2022). Transforming representations of movement from body- to world-centric space. Nature *601*, 98–104. https://doi.org/10.1038/s41586-021-04191-x.

54. Lyu, C., Abbott, L.F., and Maimon, G. (2022). Building an allocentric travelling direction signal via vector computation. Nature *601*, 92–97. https://doi.org/10.1038/s41586-021-04067-0.

55. Liang, Y., Pertzov, Y., Nicholas, J.M., Henley, S.M.D., Crutch, S., Woodward, F., Leung, K., Fox, N.C., and Husain, M. (2016). Visual short-term memory binding deficit in familial Alzheimer's disease. Cortex *78*, 150–164. https://doi.org/10.1016/j.cortex.2016.01.015.

56. Aguirre-Acevedo, D.C., Lopera, F., Henao, E., Tirado, V., Muñoz, C., Giraldo, M., Bangdiwala, S.I., Reiman, E.M., Tariot, P.N., Langbaum, J.B., et al. (2016). Cognitive decline in a Colombian kindred with autosomal dominant Alzheimer disease: a retrospective cohort study. JAMA Neurol. *73*, 431–438. https://doi.org/10.1001/JAMANEUROL.2015.4851.

57. Folstein, M.F., Folstein, S.E., and McHugh, P.R. (1975). "Mini-mental state". A practical method for grading the cognitive state of patients for the clinician. J. Psychiatr. Res. *12*, 189–198. https://doi.org/10.1016/0022-3956(75)90026-6.

58. Mioshi, E., Dawson, K., Mitchell, J., Arnold, R., and Hodges, J.R. (2006). The Addenbrooke's Cognitive Examination revised (ACE-R): a brief cognitive test battery for dementia screening. Int. J. Geriatr. Psychiatry *21*, 1078–1085. https://doi.org/10.1002/gps.1610.

59. Mulder, C., Verwey, N.A., van der Flier, W.M., Bouwman, F.H., Kok, A., van Elk, E.J., Scheltens, P., and Blankenstein, M.A. (2010). Amyloid-β(1–42), total tau, and phosphorylated tau as cerebrospinal fluid biomarkers for the diagnosis of Alzheimer disease. Clin. Chem. *56*, 248–253. https://doi.org/10.1373/clinchem.2009.130518.

60. Sun, X., and Xu, W. (2014). Fast implementation of DeLong's algorithm for comparing the areas under correlated receiver operating characteristic curves. IEEE Signal Process. Lett. *21*, 1389–1393. https://doi.org/10.1109/LSP.2014.2337313.

STAR★METHODS

KEY RESOURCES TABLE

REAGENT or RESOURCE	SOURCE	IDENTIFIER
Dataset	This paper	https://zenodo.org/record/8253734
Original code and analysis	This paper	https://zenodo.org/record/8253734

RESOURCE AVAILABILITY

Lead contact

Further information, clarification about the dataset or the code can be directed to Andrea Castegnaro (uceeaca@ucl.ac.uk). Any other request can be directed to the corresponding author, Neil Burgess (n.burgess@ucl.ac.uk).

Materials availability

The current study has not have generated any new material.

Data and code availability

- De-identified human data have been deposited at https://github.com/Lenakeiz/GenerativeLinearAngularModelPathIntegration with a snapshot deposited on Zenodo. Dataset is publicly available as of the date of publication. DOI is listed in the key resources table.
- All original code has been deposited at https://github.com/Lenakeiz/GenerativeLinearAngularModelPathIntegration with a snapshot deposited on Zenodo. The code comprises the GLAMPI model as well as all of the analysis and figures presented in the study. Instructions for how to run the code are present in the README.md file found in the root folder of the project. The code is available as of the date of publication. DOI is listed in the key resources table.
- Any additional information required to re-analyse the data reported in this paper is available from the lead contact upon request.

EXPERIMENTAL MODEL AND STUDY PARTICIPANT DETAILS

Data from healthy younger participants (n=31; 23 females; mean age 21.2, SD 3.2), healthy elderly controls (n=36; 25 females; mean age=68.3, SD 7.2) and participants with mild cognitive impairment (MCI) (n=43; 14 females; mean age 71.4, SD 8.2) were used in this study. Healthy elderly controls and the MCI groups datasets were obtained from Howett et al.[2] MCI participants were divided into three categories based on their CSF biomarker status providing evidence of underlying Alzheimer's disease (unknown = 18, negative = 14, positive = 11). A neuropsychological test battery comprising the Mini Mental State Examination[57] and the Addenbrooke Cognitive Examination Revised[58] was administered to the elderly and MCI patients for assessing their overall cognitive states (see Table S1 for details about demographics and neuropsychological tests). Threshold for positive CSF biomarker status was set as CSF amyloid <550 pg/ml, CSF tau >375 pg/ml with a CSF tau: amyloid ratio of >0.8.[59] The researchers conducting the iVR tests were blinded to the patient status, see Howett et al.[2] for more details. MCI+ and MCI- groups were age-matched and did not present any group difference in years of education (please see Table S1 for details). Note that two participants (one MCI+ and one MCI unknown) are not present from the original Howett et al.[2] dataset due to technical information loss. Healthy young participants were recruited from the UCL Sona volunteer pool and were screened prior to testing to control that they had no history of neurological or psychiatric disorders and no sensory or motor difficulties that would interfere with their capacity for VR testing. Six young controls, three healthy elderly participants, two MCI positive and two MCI unknown patients were further excluded from the dataset used in this study due to incomplete performance data or impossibility to fit the model, see data pre-processing below for details.

Ethical approval was granted by the UCL Research Ethics Committee (ID number: ShaPS-2018-JK-027). Ethics were in line with the regulations outlined in the Declaration of Helsinki (WMA, 2013).

METHOD DETAILS

Path Integration task

Each participant performed an immersive virtual reality triangle completion task to assess their ability of path integrating. The task was performed using the HTC Vive, which uses an external tracking system to track a walkable area. The elderly group was tested on trackable areas of 4.5 x 4.5 m^2 and 4.0 x 4.0 m^2, young participants were tested on a trackable area of 3.5 x 3.5 m^2, all MCI groups

were tested on a trackable area of 4.0 x 4.0 m^2 (see "outbound path additional information" for more details). The entire task was developed in the Unity game engine. Participants were guided on the outbound path of the triangle by using visual markers displayed in VR as inverted cones with a label indicating their order (Figure 1A). Participants were guided through the first three cones, displayed only one at a time. Upon reaching a given cone's location, that cone disappeared, and stereo auditory feedback prompted the participants to travel to the next newly appeared cone. Upon reaching the third and final cone, an in-VR text message was displayed instructing participants to return to the location of the first cone without further guidance (inbound path) and to confirm their estimated location by pressing a trigger on the hand-held controller.

Virtual environments did not have any enclosure or local landmarks, and boundary cues were projected at infinity. Participants were instructed to always walk in straight lines, at a constant comfortable speed, and to turn only at cone locations. To avoid participants walking beyond the tracking area, a red warning message was displayed in VR every time participants went past the tracking boundary by 30 cm. The warning message instructed participant to stop immediately, take a step back and press the response button to conclude the trial. Trials in which participants hit the limits of the tracking area were marked as "out-of-bound" trials.

Three distinct environments were displayed in the task, each featuring unique surface details (e.g., grass), boundaries (e.g., uniform mountains), and light settings (e.g., daylight). Surface details were enhanced by using detailed and realistic foliage which was reactive to wind sway to provide better optic flow to the participant.

To interrogate how different types of cues contribute to path integration, the environment was altered between the end of the outbound path and the start of the inbound path. Three environmental conditions were created: "no change" where participants always saw the full details of the environment and the outbound and inbound pathway settings were identical; "reduced optic flow" where the surface details were removed, and the ground texture was changed to a colour that merged seamlessly with the textures adopted for the boundary cues; "reduced distal cues" where all boundary cues were removed (see Figure S1A). A screen fading to black within 1 seconds was used to temporarily occlude the view to implement changes in the environment.

Participants completed a total of 27 (36 in the case of the young control group) trials. For each of the three different environments, participants completed the inbound path in three distinct environmental conditions ("no change", "reduced optic flow", "reduced distal cue"), and there were 3 (4 for young controls) trials in each condition within each environment. Each environment was visited within an experimental block. The order of the environment and the conditions within the environment were shuffled at the start of the experiment to minimise the use of environment-dependent strategies and maximise reliance on self-motion cues.

The triangle path was generated pseudo-randomly by an in-house algorithm at the beginning of each trial within the tracking area set for the HTC Vive. The algorithm assigned the location of cone 1 in the proximity of the furthest corner from the participant's location at the end of the previous trial.

The other cone locations (2-3) were determined by choosing locations in the proximity of each of the opposite sides from the corner where cone 1 was placed. The algorithm ensured that cones 2 and 3 were never close together by excluding distances between cone 2 and 3 smaller than one meter. The pseudo-random algorithm was designed this way to maximise the amount of walking space available. Distribution of triangle path lengths (l_1 and l_2) and turning angles (θ_2) generated this way are reported in the histogram plot in Figure S6.

For each trial, the following information was saved as an output: the locations of the generated cones and timestamps when they appeared, the timestamps of participant reaching each cone, the location and timestamp of the participant's response (their estimation of cone 1 location), whether the participant hit the out of boundary area during the trial, and the position and orientation (quaternion) of the participant headset if/when they hit the out of boundary. Additionally, for each trial the participant headset position and orientation were constantly tracked at 10 Hz (100 ms).

Data pre-processing

Given that participants performed different shapes of outbound pathways due to the pseudo-random generation of the cone locations, we standardised the outbound pathways in the Cartesian coordinate space through a linear transformation (please refer to Figure 1B as an example of a pre-processed trial). We relocated the locations of the first cone to the coordinate origin (0,0) and aligned the first outbound pathways with the positive x-axis. Furthermore, whenever the outbound turn between the first and second segment was to the right, this turn was flipped to the left, such that all trials had the vector representing the second outbound path point towards the II quadrant of the Cartesian plane (please see Figure 2A for a schematic of the intended result).

We previously reported ~30% of the trials were out-of-bound trials in the original dataset.[2] Since participants were instructed to stop immediately upon walking beyond the tracking area and the trial was subsequently terminated, the out-of-bound trials did not provide representative distance information. However, angular information was obtained before the trial termination, and therefore remains valid and informative in the out-of-bound trials. Therefore, we decided to only discard the distance information rather than the whole trial and incorporated the angle information into the model fitting process. Out-of-bound angles were calculated between the initial direction at the start of the inbound path (indexed by a vector pointing from the second to the third cone) and the direction at the location at the time of hitting the boundary (indexed by a vector pointing from the third cone to the location of hitting the boundary). We excluded six young controls, three healthy elderly and one participant with unknown CSF status, due to a tracking system fault that prevented the collection of location data at the time they hit the boundary.

Additionally, we excluded trials in which participants did not show a clear intention of movement direction by excluding trials for which the inbound path was terminated within 0.5m of the start. Finally, tracking data for all trials was visually screened to ensure that participants complied with the instructions (e.g., do not retrace the outbound path for estimating the location of cone 1).

According to these criteria, excluded trials totalled to 253 (22.7%) for the young group, 84 trials (8.6%) for the healthy elderly group, 54 trials (11.1%) for the MCI unknown group, 9 trials (2.4%) for the MCI negative group and 30 trials (10.1%) for the MCI positive group. From the remaining trials, the included out-of-bound trials accounted for 13.8% in the young group (calculated on the first 9 trials only, see path integration task section), 27.8% in the healthy elderly group, 32.6% in the MCI unknown group, 29.7% in the MCI negative group, 29.3% in the MCI positive group. We also calculated the ratio of out-of-bound trials for each participant and reported them in Figure S1B.

To prevent overfitting in parameter estimation, we did not carry out the fitting process if the number of trials completed was less than the number of parameters (i.e., 7). This step didn't affect the parameter fitting for young, healthy elderly and MCI negative group under any environmental condition. From the MCI group with positive biomarkers, two participants have been excluded under all of the conditions and one participant under no change condition. From the MCI with biomarker status unknown one participant has been excluded under all three conditions and one participant under reduced distal cue condition. Since we excluded these data from model estimates, for a fair comparison between behavioural data and model estimates, we also excluded these data for the behavioural analysis (Figures 1 and 6).

The pre-processing methodology employed in the present study diverges from that of Howett et al.,[2] where out-of-bounds trials were simply omitted, and no additional criteria were set.

Calculation of path integration error

To interrogate path integration performance in more detail, the final error was split into its linear (distance) and angular components. Computing proportional, rather than absolute, distance and angular errors accounted for the variability in the probed triangle paths within and between participants stemming from the pseudo-random generation of triangle shapes. For each trial, proportional linear error was a ratio between the length of the performed inbound path (the length of the vector from the third cone to the participant's response) and the length of the correct inbound path (the length of the vector from the third cone to the first cone). The proportional angular error was a ratio between the angle between the final direction at the end of the outbound path (vector from the second to the third cone) and the direction at the location of participant's response (vector from the third cone to the participant's estimated location) and the correct inbound angle (computed to the location of the first cone, rather than participant's estimation of it). We calculated the egocentric participant's turn wrapped in the range of $[0, 2\pi]$, assuming that the intended response was always made using the short (anti-clockwise) turn to complete the triangle from cone 3.

A two-way ANOVA was performed to analyse the effect of group (healthy young participants, healthy elderly participants, MCI unknown, MCI negative, MCI positive patients) and the effect of the inbound condition (no change, reduced optic flow, reduced distal cues) on the calculated behavioural measures.

The computational model

We built a generative model of the inbound path to better understand the source of errors in the PI task. Instead of being built as a homing vector model, our model is a configural model which predicts both the inbound distance and angle by considering the effects of encoding, calculation, and production errors in both walking distance and turning angles, as well as the effects of distance and angular noises. We define the actual walked distance in two outbound segments as l_1 and l_2, respectively, and the actual turning angle between the two segments as θ_2. It is worth noting that θ_2 was calculated after flipping all the right turn trials to left turn trials (see data pre-processing section for details). This approach allowed us to ensure that the angular turn was always representative of the egocentric rotation (range $[-\pi; \pi]$) and was consistent across trials.

Since participants were guided by visual markers during outbound walking, we assume that distance execution is perfect, and there are only encoding errors to accumulate during this stage. This distance encoding is modelled by a leaky integrator $v(t)$ of the walking speed over time, which is written as:

$$\frac{d\,l'(t)}{dt} = -\beta l'(t) + kv(t). \quad \text{(Equation 1)}$$

Here $l'(t)$ is the encoded distance at time t, β is a forgetting term (or leaky integration) with $\beta > 0$ ($\beta < 0$ would represent growth of the encoded walked distance), and k is the speed gain, k=1 representing that the speed is converted perfectly into the instantaneous walking distance. Assuming that the walking speed on an outbound path is constant (but can be different across trials), the encoded distance can be written as (see below for the derivation):

$$\begin{cases} l_1' = l_1 \dfrac{k\left(1 - e^{-\beta T_1}\right)}{\beta T_1} e^{-\beta T_2}, \\ l_2' = l_2 \dfrac{k\left(1 - e^{-\beta T_2}\right)}{\beta T_2}. \end{cases} \quad \text{(Equation 2)}$$

Here T_1 and T_2 are the time spent on the two outbound paths, respectively. Note that since l_1 takes place before l_2, encoding error in the first segment continued to accumulate when participants walked on the second segment (by multiplying $e^{-\beta T_2}$), which has been indicated in Harootonian et al.[13] It is also noteworthy that Equation 2 shows that the encoded distance depends on the time spent on the path, i.e., the longer the time that participants spent on the path (the slower the participants walked), the greater the encoding

CellPress
OPEN ACCESS

error. In other words, our model captures the variability in walking duration across trials. This is different from the leaky integrator model used in desktop VR path integration tasks, which assumes that distance encoding is independent from the walking speed or the time taken (see "relationship with the leaky-integrator model used in desktop VR" and Figure S2B for more details).

As with the guided walking on the outbound path, we consider that the encoded turning angle is simply implemented by multiplying the physical turning angle with a gain factor g_2, which gives:

$$\theta_2' = g_2\theta_2. \quad \text{(Equation 3)}$$

$g_2 < 1$ represents an under-estimation of the turning angle, whereas $g_2 > 1$ represents an over-estimation of the turning angle. Based on the encoded distance l_1', l_2' and angle θ_2', participants are assumed to calculate the intended inbound distance h and angle α as

$$\begin{cases} h = \sqrt{\left(l_1' + l_2' \cos\theta_2'\right)^2 + \left(l_2' \sin\theta_2'\right)^2} + \xi_d, \\ \alpha = \pi - \sin^{-1}\left(\dfrac{l_1' \sin\theta_2'}{h}\right) + \xi_a. \end{cases} \quad \text{(Equation 4)}$$

Here ξ_d and ξ_a are two noise terms, representing inaccurate calculation error, which we model as part of the Gaussian noises described below.

Finally, we include an additional type of error in the PI task, namely the production error, characterised by a regression-to-the-mean effect in producing the distance/angle from the intended inbound distance and angle, which are:

$$\begin{cases} l_3' = m_3 h + (1 - m_3)\bar{l}_r, \\ \theta_3' = g_3\alpha + (1 - g_3)\bar{\theta}_r. \end{cases} \quad \text{(Equation 5)}$$

Here l_3' and θ_3' the produced inbound distance and angle (predicted values from the generative model), respectively. m_3 and g_3 are the regression slopes, with $\bar{l}_r$ and $\bar{\theta}_r$ the corresponding averaged (across all trials) correct inbound distances and egocentric angles (range [-2π, 2π]), respectively. For instance, if $0 < g_3 < 1$, the angular production has the effect of overturning an intended angle which is smaller than $\bar{\theta}_r$, as well as under-turning an intended angle which is larger than $\bar{\theta}_r$ (see Figure 2B). Since participants experience a sequence of several trials in the PI task, such regression-to-the-mean effect can also be seen as how much they remember the mean inbound distance or angle. For instance, a g_3 close to zero indicates that no matter what the intended angle is, the produced angle is always near $\bar{\theta}_r$ (remembering the mean angle), whereas a g_3 close to 1 indicates the tendency to produce an angle like the intended one, without regard to the mean angle (forgetting the mean angle). Since participants are suboptimal in many stages of the triangle completion task, and noise can occur anywhere (not just in the calculation errors described above), we modelled this by assuming that the actual inbound distance l_3 and angle θ_3 are sampled from two Gaussian distributions centred at the predicted distance l_3' and angle θ_3' from the model, respectively, which are:

$$\begin{cases} p\left(l_3 \middle| l_3'; \Theta\right) = \dfrac{1}{\sqrt{2\pi}\sigma} exp\left(-\dfrac{\left(l_3 - l_3'\right)^2}{2\sigma^2}\right), \\ p\left(\theta_3 \middle| \theta_3'; \Phi\right) = \dfrac{1}{\sqrt{2\pi}\nu} exp\left(-\dfrac{\left(\theta_3 - \theta_3'\right)^2}{2\nu^2}\right). \end{cases} \quad \text{(Equation 6)}$$

σ reflects the variance in the generation of l_3, and ν reflects the variance in the generation of θ_3. Since the calculation errors are now treated as part of the Gaussian noises, we simply set $\xi_d = \xi_a = 0$ (described above) $\Theta = \{\beta, k, g_2, m_3, \sigma\}$ defines the parameter set for generating the probability distribution for l_3, $\Phi = \{\beta, k, g_2, g_3, \nu\}$ defines the parameter set for generating the probability distribution for θ_3. To estimate these parameters for each participant, we compute the negative log-likelihood of the data (i.e. l_3, θ_3 for each trial) as the product of the joint probability distribution (Equation 6) summed over all trials, which gives:

$$NLL_i(\Theta_i \cup \Phi_i) = \sum_{j=1}^{N_i} \frac{\left(l_{3,j} - l_{3,j}'\right)^2}{2\sigma} + \sum_{j=1}^{N_i} \frac{\left(\theta_{3,j} - \theta_{3,j}'\right)^2}{2\nu} + N_i \log\sigma + N_i \log\nu + N_i \log 2\pi, \quad \text{(Equation 7)}$$

where i is the participant index, and N_i the total number of trials. It is important to note that the likelihood for distance was excluded in out-of-boundary trials due to the unavailability of the participants' intended stop location. We then find the best configuration of parameters by minimizing the negative log-likelihood with MATLAB's *fmincon* with *GlobalSearch* option which aims to find the global minima in the parameter space for this non-linear optimization problem (see above for a link to the Github repository).

Outbound path additional information

Participants in the path integration task were recruited from various sites between Cambridge (elderly and individuals with mild cognitive impairment) and London (young controls). Due to the availability of testing facilities at each site, the maximum trackable area for safe movements varied between different participant groups. As the generation of triangle paths depended on the trackable area, each group experienced different path lengths. To investigate behavioural differences in the outbound path across the groups,

we calculated various measures including the average length, the average time spent and the average walking speed. Additionally, we accounted for potential variations in vestibular perception due to postural differences by extracting the pitch angle, which represents the angle of the face direction with the world up axis.[35] Speed information was reconstructed from tracking data acquired at a rate of 10 Hz, processed as follows. First, the tracking data was filtered using the timestamps when participants reached each cone to include only tracking data from the outbound path. Next, a moving average speed was calculated to smooth out fluctuations in tracking measurements using the following:

$$s_i = \alpha s_{i-1} + (1-\alpha)\frac{\|x_i - x_{i-1}\|}{t_i - t_{i-1}}, \quad \text{(Equation 8)}$$

where α is set to 0.9, x_i is the tracked position and t_i is the recorded time at the same index. The reconstructed speed was further smoothed by convolving with a Gaussian kernel with a width equalling to 1-second. The average speed of the participant was then calculated by integrating the reconstructed speed curve, with stationary periods being excluded using a threshold of 0.2 m/s. Pitch angle was extracted between the timestamps of reaching cone two and cone three to isolate the posture effects on the turn of the outbound path.

To assess differences between the group means, a one-way ANOVA was performed in each of the extracted measures. Post hoc comparisons were conducted using Bonferroni corrections. Younger controls who were tested in a smaller room setup experienced smaller triangles as compared to the healthy elderly participants (Figure S1C; F(4,94)=73.9, p<0.001). As a result, young participants spent on average less time (Figure S1D; F(4,94)=9.15, p<0.001) and walked slower (Figure S1E; F(4, 105)=8.68, p=0.003) than elderly participants. There were no significant differences in the overall behaviour during the outbound path between healthy elderly participants and the MCI groups, except for the duration of the outbound path. Healthy elderly participants spent more time on average during the outbound path compared to the MCI unknown group (Figure S1D; F(4, 94)=9.15, p = 0.006). These differences are not ideal, but we note that our behavioural measures (ratios indicating under-walking and overturning) and model parameters are not proportional to the overall scale. In addition, the longer paths taken by the healthy elderly, if anything, allow more scope for error and so work against our main findings of impairments in MCI relative to the healthy elderly, and absence of impairment in elderly relative to younger participants.

For completeness, it is worth noting that younger participants experienced shorter outbound paths (all p's < 0.001) and slower walking speeds compared to each of the MCI groups (all p's < 0.05). Furthermore, the mean pitch angle varied significantly between the young and MCI negative group (F(4, 105)=6.08, p < 0.001), with the latter group demonstrating a tendency to orient their heads towards the ground. It should be noted, however, that these comparisons were not the primary focus of our study.

Deriving the distance encoding component from a leaky-integrator model

The leaky integrator model we considered is given as follows:

$$\frac{d\,l'(t)}{dt} = -\beta l'(t) + kv(t), \quad \text{(Equation 9)}$$

where $l'(t)$ is the encoded distance which is reduced proportionally to its current value (leaky) and incremented by the walking speed (integration). β is the rate of memory decay (if $\beta > 0$; otherwise, it represents the rate of memory gain, but for convenience of description, we term the above equation as a leaky integrator). k is a speed gain, with k=1 representing that the speed is converted perfectly into the instantaneous walking distance. The equation represents that participants continuously update their internal estimation of traversed distance by using an estimation of their walking speed.

By assuming that a participant's walking speed is constant on a given outbound path (the speed may vary across trials), then, a general solution to Equation 9 is:

$$l'(t) = e^{-\beta t + c} + \frac{kv}{\beta}, \quad \text{(Equation 10)}$$

where v is a constant, and c is obtained by the limits of integration, for which $l'(0) = 0$. Therefore:

$$e^c + \frac{kv}{\beta} = 0, \quad \text{(Equation 11)}$$

which gives $c = \ln\left(-\frac{kv}{\beta}\right)$. Thus, the full solution to Equation 9 is given by:

$$l'(t) = e^{-\beta t + \ln\left(-\frac{kv}{\beta}\right)} + \frac{kv}{\beta} = \frac{kv(1 - e^{-\beta t})}{\beta}. \quad \text{(Equation 12)}$$

Since $v = \frac{l(t)}{t}$, Equation 12 can be re-written as:

$$l'(t) = \frac{l(t)k(1 - e^{-\beta t})}{\beta t}. \quad \text{(Equation 13)}$$

CellPress
OPEN ACCESS

Therefore, when participants finish walking the second outbound leg, the length of the second leg is encoded as:

$$l'_2 = l_2 \frac{k(1 - e^{-\beta T_2})}{\beta T_2}, \quad \text{(Equation 14)}$$

with l_2 the actual walking distance and T_2 the total time spent on the second outbound leg. We further assume that the encoded distance of the first leg keeps updating when participants walk on the second leg, and thus, the distance of the first leg is encoded as:

$$l'_1 = l_1 \frac{k(1 - e^{-\beta T_1})}{\beta T_1} e^{-\beta T_2}. \quad \text{(Equation 15)}$$

with l_1 the actual walking distance and T_1 the total time spent on the first outbound leg. $e^{-\beta T_2}$ represents the exponential decay of the memorized walking distance on the first leg after participants spend T_2 on the second leg. Equations 14 and 15 show the encoded distance used in our generative model (see Equation 2).

Relationship with the leaky-integrator model used in desktop VR

In previous desktop-VR-based path integration tasks[14,19] where participants provide inputs using a joystick, a leaky-integrator model without considering walking speed was used as follows:

$$\frac{dl'}{dl} = -\alpha l' + k, \quad \text{(Equation 16)}$$

where l is the actual navigated distance in the VR environment and l' is the encoded distance. α is the rate of decay of the integrator (for $\alpha > 0$), and k is the gain of the visual sensory input, with $k = 1$ a perfect transformation of the optical flow into the instantaneous travel distance. Equation 16 shows that in each step dl, the encoded distance dl' is reduced proportionally to its current value and incremented by the distance given by the gain k of the step. Solving Equation 16 as we described in Equations 9, 10, 11, and 12 gives:

$$l' = \frac{k}{\alpha}(1 - e^{-\alpha l}). \quad \text{(Equation 17)}$$

Here we establish the link between the path integrator in the immersive VR and the path integrator in the desktop VR. Since $dl = vdt$, dividing both side of Equation 9 by v, we obtain:

$$\frac{d\,l'}{dl} = -\frac{\beta}{v} l'(t) + k. \quad \text{(Equation 18)}$$

If we assume $\alpha = \beta / v$, then Equation 18 describes the same dynamics as given in Equation 16. However, Equation 16 indicates that no matter how fast participants move in the desktop VR environment, as long as the actual navigated distance is the same, the internal encoded distance will be the same. On the contrary, Equation 18 (also reflected in Equations 14 and 15) indicates that as participants move faster, the memory decay rate will be reduced accordingly, and they will show less forgetting of the walked distance (note that in the original Equation 9, the actual memory decay rate is independent of the walking speed, but the effect of less elapsed time associated with greater speed to traverse the same distance will be the same as a reduced decay rate showed in Equation 16). It will be interesting to further explore in future experiments whether the encoded distance is affected by the walking speed in real-world PI tasks.

Correlation between model parameters and age or educational duration

To show that the parameter differences in our model are not confounded with demographic variables such as age and education, we checked the age and educational duration differences between MCI+ and MCI- by running two-sample t-tests (see Table S1). These results showed that there was no statistical difference in age (t(23)=0.200), as well as no statistical difference in educational duration (t(22)=0.962) between the MCI+ and MCI- groups. Moreover, we also tested the correlation between these potential confounds and estimated parameter values (e.g., g_2, ν) in the healthy elderly participants. None of them show significant correlations: age v.s. g_2: r=0.22, p=0.227 (no change); r=0.04, p=0.842 (reduced distal cue); r=-0.09, p=0.625 (reduced optical flow); age v.s. ν: r=0.09, p=0.631 (no change); r=0.11, p=0.554 (reduced distal cue); r=0.0, p=0.991 (reduced optical flow). Overall, MCI+ and MCI- groups were age-matched and did not present any group difference in years of education, and neither age nor years of education correlated with any GLAMPI parameters within the healthy elderly participants.

QUANTIFICATION AND STATISTICAL ANALYSIS

Two-way ANOVA was employed to investigate the main effects and interactions of ’group’, either young, healthy elderly and pooled MCI or MCI+ and MCI-, and ’environmental conditions’ (including no change, reduced optic flow and reduced distal cues) on behavioural metrics or the GLAMPI parameters. Post hoc comparisons to assess differences between groups were Bonferroni corrected. Post hoc statistical power analysis was performed on significant results, by calculating the effect size and achieved power using the G*Power software. GLAMPI fitted parameters were compared to their respective nominal values using two tailed t-tests.[31]

To assess the ability of individual GLAMPI model parameters to discriminate the healthy elderly from the pooled MCI group, as well as the MCI+ from the MCI-, we used a support vector machine classification using a linear kernel with a hold-out strategy of the training set constituting 60% of the total sample. This was an appropriate choice for testing individual parameters, allowing the support vector machine to find a threshold separating the groups. We created the Receiver Operating Characteristic (ROC) using the posterior probabilities obtained from the model prediction on the remaining testing set. A total of 1000 repetitions was used and for each repetition the associated area under the curve (AUC) was calculated. The analysis was performed for each of the fitted value of the GLAMPI model parameters. Finally, to enable comparisons between our mathematical model and the path integration task we run the same analysis on the path integration behavioural performance, i.e., the proportional linear error and the proportional angular error. To statistically assess the differences between the ROC curves, we conducted DeLong's test,[60] comparing the AUCs between different classification outcomes. Since the ROC curves are cross-validated, we adopted two ways to perform DeLong's test. First, we perform DeLong's test for each repetition, which results in 1000 p-values in total and we calculate the percentages that show significant differences in the AUC values. Second, we averaged the posterior probabilities (one for behavioral data and one for the model parameter) on heldout data over the 1000 repetitions and performed DeLong's test on the mean groups.

Current Biology, Volume *33*

Supplemental Information

Overestimation in angular path integration precedes Alzheimer's dementia

Andrea Castegnaro, Zilong Ji, Katarzyna Rudzka, Dennis Chan, and Neil Burgess

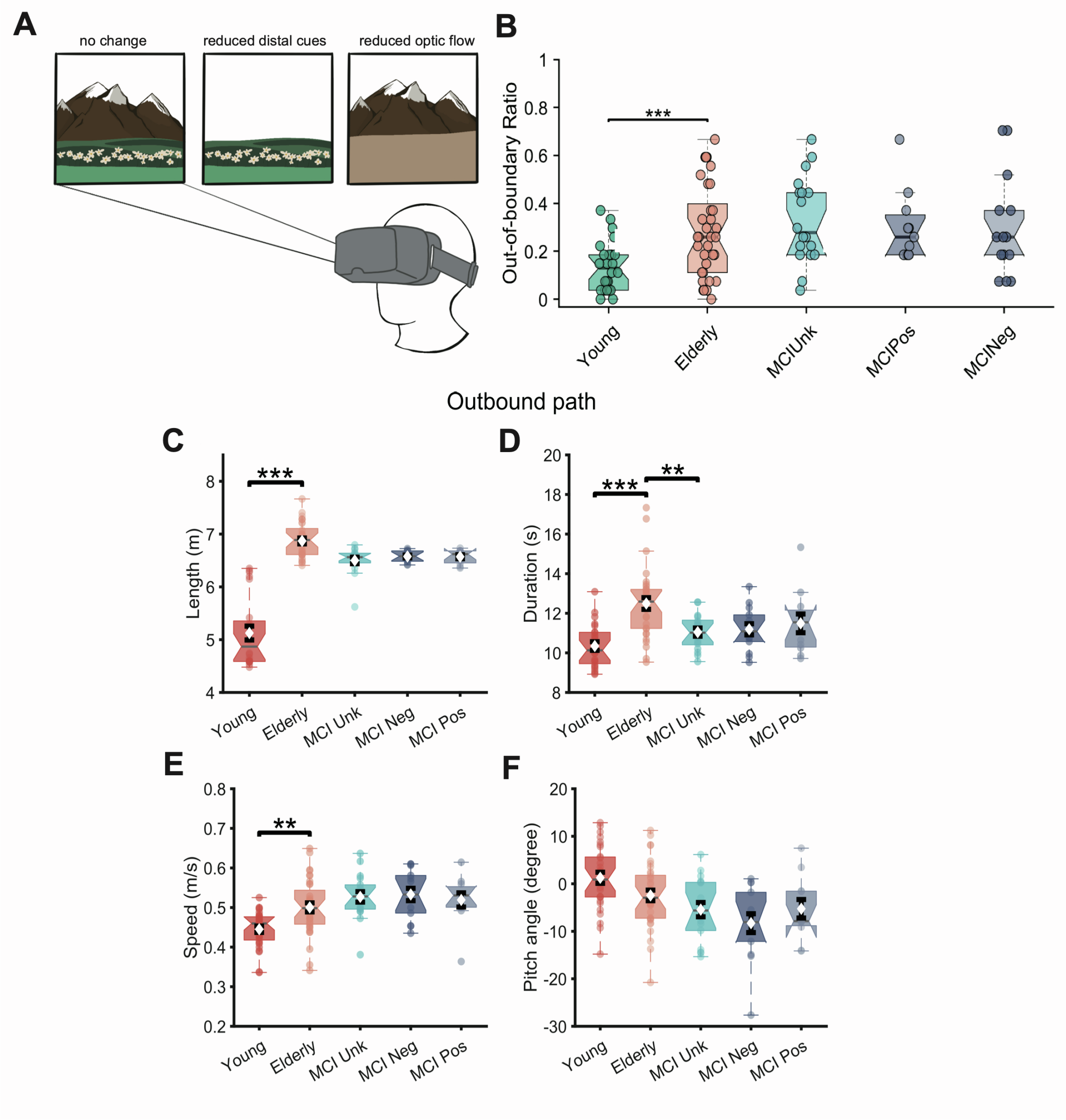

Figure S1: Experimental setup and behavioral results. Related to Figure 1 and STAR Methods. *(A) Inbound path environmental conditions. Following completion of the outbound path, participants were presented with three distinct environmental conditions in a randomized order just prior to starting the return inbound path. Environmental conditions were applied with a fading to black transition in the virtual reality view. The "No Change" condition consisted of an unchanged environment as compared to the outbound path. The "Reduced Distal Cues" condition involved the removal of distant landmarks from the scene. The "Reduced Optic Flow" condition involved the removal of details from the ground plane and replacement with a solid-colored plane merging seamlessly with the distant cues. (B) out-of-boundary ratios across groups. A two-way ANOVA analyzed the effect of environmental conditions (no change, reduced optic flow, reduced distal cues) and participant group (young vs. healthy elder participants vs. MCIUnk vs. MCIPos vs. MCINeg patients) on the out-of-boundary ratios. There was no significant interaction effect (F(8,285)=0.29, p=0.931), no significant environmental effect (F(2,285)=0.21, p=0.936). There was a significant group effect with F(4,285)=8.64, and p<0.001. Post-hoc Bonferroni corrected comparisons showed that the young group made significantly less out-of-boundary trials than the other four groups: young vs elderly p<0.001; young vs MCIUnk p<0.001; young vs MCIPos p<0.001; young vs MCINeg p=0.002. (C-F) outbound path information for different groups. (C) average length of the outbound path (sum of* l_1 *and* l_2*). (D) average time that to complete the outbound path. (E) reconstructed average walking speed during the outbound path. Each dot represents the average value of the participant. (F) average pitch angle obtained from the headset orientation. Filled diamonds represent the group mean with black error bars indicating the standard error of the mean (SEM). The indent and gray horizontal bar in each box represents the median value. The bottom and top of each box*

*represent the 25th and 75th percentiles, respectively. Horizontal bars indicate significant post-hoc comparisons. Differences reported are between young and healthy older and between healthy older and any of the MCI groups. The significance of comparisons between young and MCI subgroups were not marked on figures for legibility and are not referred to in the main text. * ($p<0.05$), **($p < 0.01$), ***($p < 0.001$) are marked from the two-way ANOVA with post hoc multiple comparisons with Bonferroni correction.*

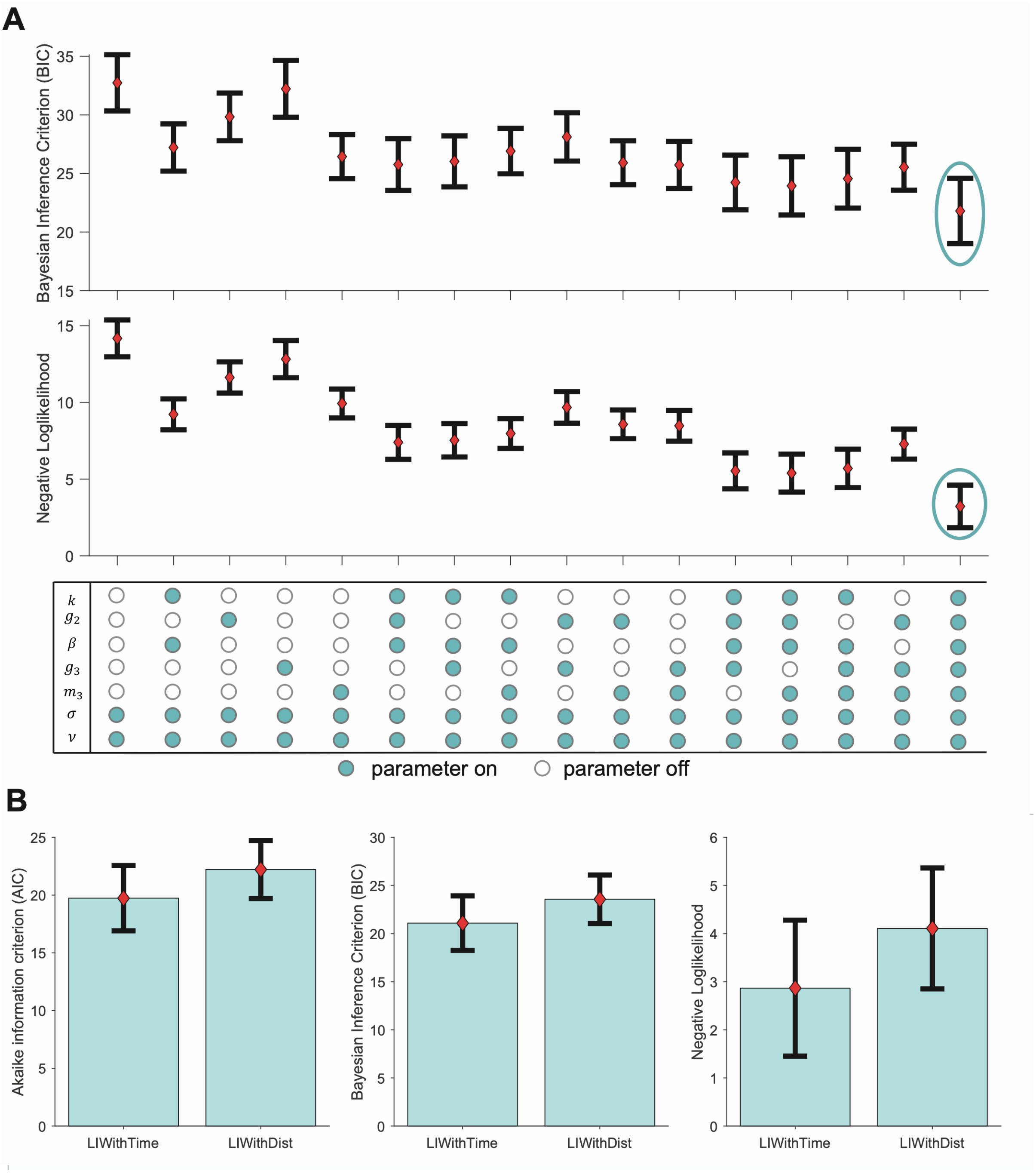

Figure S2: GLAMPI model selection. Related to Figure 2. *(A) Comparison between different configurations of the model and the relative Bayesian Information Criterion (BIC) and negative loglikelihood value (NLL). Top: BIC values (mean with SEM) of candidate models. Middle: NLL values (mean with SEM) of candidate models. Bottom: blue/white circles represent considered/unconsidered parameters. Candidate models were created from all different combinations of models varying type and the number of source errors used. The selected model with lowest BIC or NLL values are circled in blue. (B) comparison between leaky integration over time and leaky integration over distance. It shows the model performance of AIC BIC and loglikelihood of the two models on the data from healthy elderly participants. Leaky integration of distance is described in the section '*Relationship with the leaky-integration model used in desktop VR*'. We found that leaky integration over time has a better performance than leaky integration over distance, reflected in the lower AIC BIC and negative Loglikelihood values. However, the Wilcoxon rank sum test shows that the AIC, BIC and negative Loglikelihood values are not significantly different from each other in these two models.*

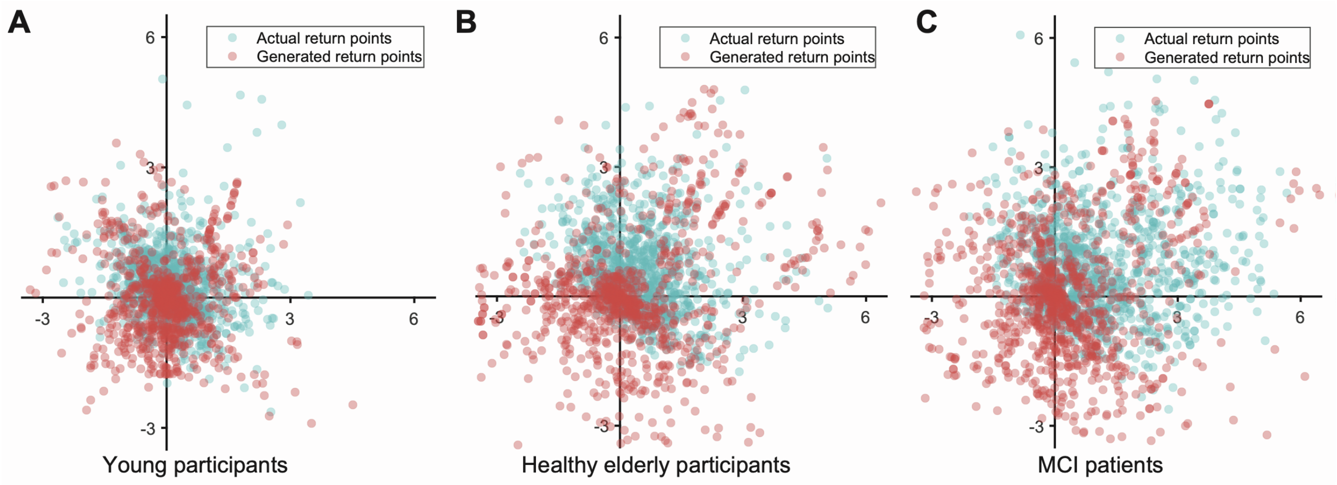

Figure S3: Summarized actual return locations and generated return locations. Related to Figure 3. *Showing young participants, healthy elderly participants, and MCI patients. Blue dots: actual return locations (each for one trial). Red dots: predicted return locations from the model.*

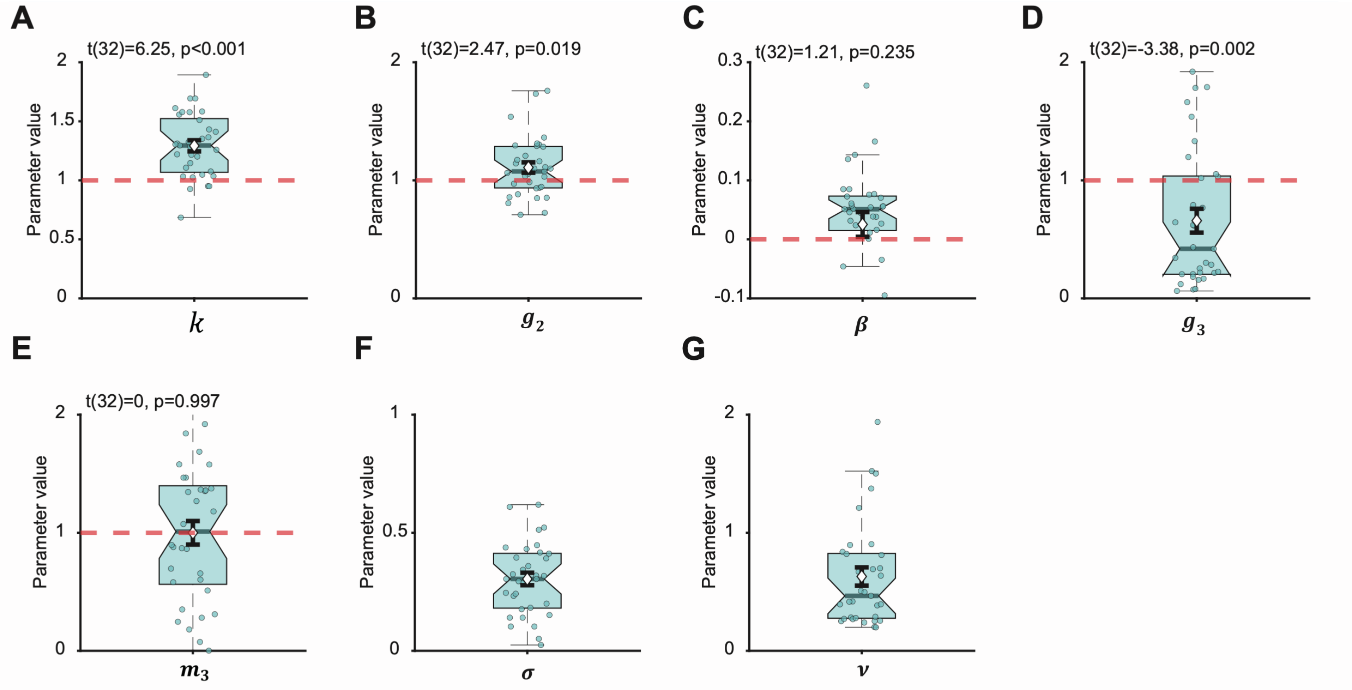

Figure S4: Estimated parameters of the GLAMPI model on the data from healthy elderly participants. Related to Figure 4. *Showing the significance of each parameter's difference from its optimal value (red dashed line) at which no error would occur according to one sample two-tailed t-test results. Each dot represents a parameter estimated from the data of one participant (averaged across three conditions). Box plots shown as in Figure S1.*

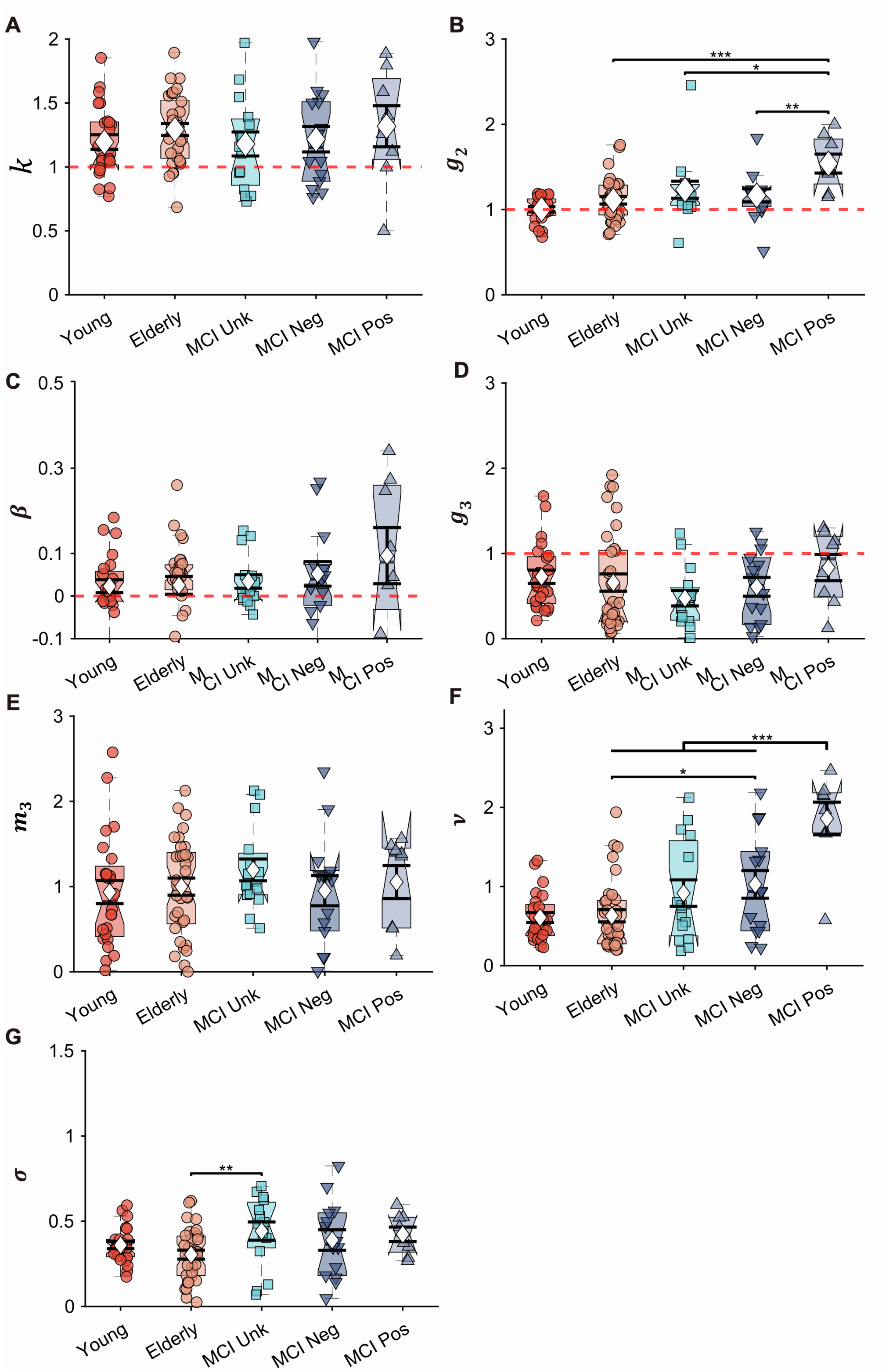

Figure S5: GLAMPI parameter comparisons among young, healthy elderly participants, MCI unknown, MCI negative and MCI positive. Related to Figure 4. *Two-way ANOVA on the GLAMPI parameters with environmental condition and group as factors. Each dot represents a parameter estimated from the data of one participant (averaged across three conditions). In each plotted box, the white diamond marks the mean, the black bars mark the standard error of the mean (SEM) and the grey horizontal bar marks the median. The bottom and top of each box mark the 25th and 75th percentiles, respectively. Observations beyond the whisker length are outliers. Red dashed lines (reference lines) mark the optimal parameter value (i.e., no error occurs at this value). The significance of comparisons between young and MCI subgroups were not marked on figures for legibility and because these contrasts are not referred to throughout the manuscript. * ($p<0.05$), **($p < 0.01$), ***($p < 0.001$) are marked from the two-way ANOVA with post hoc multiple comparisons with Bonferroni correction.*

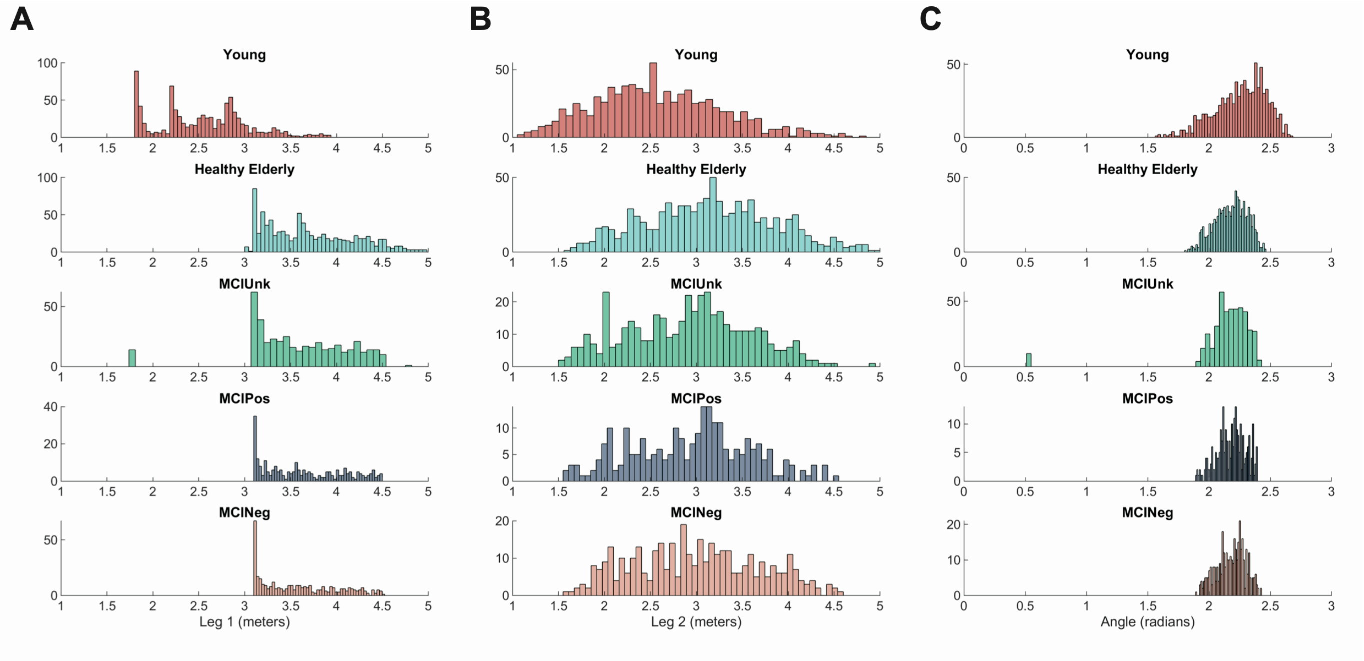

Figure S6: Histograms of the outbound paths for each group. Related to STAR Methods. *(A) The distribution of* l_1*; (B) The distribution of* l_2*; (C) The distribution of the turning angle (*θ_2*) between* l_1 *and* l_2*.*

Group	Sample Size	Age (Mean ± SD)	Sex (Female %)	Years in Ed (Mean ± SD)	ACE-R (Mean ± SD)	MMSE (Mean ± SD)
Young	31	21.16 (3.17)	23 (74.19%)	14.77 (1.21)		
Elderly (controls)	36	68.31 (7.19)	26 (69.44%)	15.08 (3.74)	97.21 (3.29)	29.74 (0.57)
MCI Negative	11	71.29 (9.09)	4 (28.57%)	14.54 (4.41)	86.69 (7.62)	27.62 (2.66)
MCI Positive	14	75.73 (7.30)	3 (27.27%)	14.45 (4.01)	82.82 (10.04)	25.82 (5.21)
MCI Unknown	18	71.22 (8.28)	7 (38.89%)	14.33 (3.43)	89.71 (5.43)	28.41 (1.58)

Table S1: Demographics and neuropsychological tests (where collected). Related to STAR Methods. *Reported data are means and standard deviations. Neuropsychological tests reported are the Addenbrooke's Cognitive Examination-Revised (ACE-R)*[S1] *and the Mini Mental State Examination (MMSE)*[S2]*. We carried out two-sample tests to check if there is any difference in the two demographic variable between MCI+ and MCI- patients. It showed that there is no significant age difference between these two groups (t(23)=0.200), and no significant difference in educational duration (t(22)=0.962). Note that all data was been tested for normality with the Anderson-Darling test before carrying out the t-test.*

Supplemental References

S1. Mioshi, E., Dawson, K., Mitchell, J., Arnold, R. & Hodges, J. R. The Addenbrooke's Cognitive Examination revised (ACE-R): A brief cognitive test battery for dementia screening. *Int J Geriatr Psychiatry* **21**, 1078–1085 (2006).

S2. Folstein, M. F., Folstein, S. E. & McHugh, P. R. 'Mini-mental state'. A practical method for grading the cognitive state of patients for the clinician. *J Psychiatr Res* **12**, 189–198 (1975).
